# Supplementary material for: Circumstances and factors of sleep-related sudden infancy deaths in Japan
Source: PLoS One. 2020 Aug 21;15(8):e0233253. doi: 10.1371/journal.pone.0233253 (PMC7444554; doi:10.1371/journal.pone.0233253)
Supplement: Attachment — Submitted filename: PONE-D-20-12699_R1_reviewer.pdf [file pone.0233253.s003.pdf]

PLOS ONE

Circumstances and factors of sudden infant deaths during sleep in Japan

--Manuscript Draft--

**Manuscript Number:**	PONE-D-20-12699R1
**Article Type:**	Research Article
**Full Title:**	Circumstances and factors of sudden infant deaths during sleep in Japan
**Short Title:**	SUDI during sleep in Japan
**Corresponding Author:**	Motoki Osawa<br>Tokai University School of Medicine<br>Isehara, Kanagawa JAPAN
**Keywords:**	Sudden infant death syndrome; Forensic pathology; Epidemiology; Pediatrics; Population data; risk factor
**Abstract:**	Background: Sudden unexpected death in infancy (SUDI) comprises both natural and unnatural causes of death. However, a few epidemiological surveys have investigated SUDI in Japan.<br>Objective: This retrospective study was conducted to investigate the latest trends of circumstances and risk factors of SUDI cases in which collapse occurred during sleep.<br>Methods: Forensic pathology sections from eight universities participated in the selection of subjects from 2013 to 2018. Data obtained from the checklist form were analyzed based on information at postmortem.<br>Results: There were 259 SUDI cases consisting of 145 male infants and 114 female infants with a mean birth weight of 2888 ± 553 and 2750 ± 370 g, respectively. Deaths most frequently occurred among infants at 1 month of age (18%). According to population data as the control, the odds ratio (95% confidence interval) of mother's age ≤19 years was 11.1 (6.9–17.7) compared with ages 30–39. The odds ratio for the fourth- and later born infants was 5.2 (3.4–7.9) compared with the frequency of first-born infants. The most frequent time of day for discovery was between 7 and 8 o'clock, and the time difference from the last seen alive was a mean of 4.1 h. Co-sleeping was recorded for 61%, and the prone position was found for 40% of cases at discovery. Mother's smoking habit exhibited an adds ratio of 4.5 (2.9–5.8).<br>Conclusion: This study confirmed the trends that have been observed for sudden infant death syndrome; particularly, very high odds ratios were evident for teenage mothers and later birth order in comparison with those in other developed countries. Neglect was suspected in some cases of the prolonged time to discovery of unreactive infants. To our knowledge, this is the first report of an extensive survey of SUDI during sleep in Japan.
**Order of Authors:**	Motoki Osawa
	Yasuhiro Ueno
	Noriaki Ikeda
	Kazuya Ikematsu
	Takuma Yamamoto
	Wataru Irie
	Shuji Kozawa
	Hirokazu Kotani
	Hideki Hamayasu
	Takehiko Murase
	Keita Shingu
	Marie Sugimoto
	Ryoko Nagao

Powered by Editorial Manager® and ProduXion Manager® from Aries Systems Corporation

	Yu Kakimoto
**Response to Reviewers:**	The detailed review of our article is appreciated. The comments by the reviewer have been helpful in allowing us to revise the manuscript. The authors have attempted to address the questions raised as separate pages. According to the raised comments, the manuscript has been rewritten extensively to a revised version. Alterations are indicated as track changes in the revised manuscript.
**Additional Information:**	
**Question**	**Response**
**Financial Disclosure**<br><br>Enter a financial disclosure statement that describes the sources of funding for the work included in this submission. Review the submission guidelines for detailed requirements. View published research articles from *PLOS ONE* for specific examples.<br><br>This statement is required for submission and **will appear in the published article** if the submission is accepted. Please make sure it is accurate.<br><br>**Unfunded studies**<br>Enter: *The author(s) received no specific funding for this work.*<br><br>**Funded studies**<br>Enter a statement with the following details:<br>• Initials of the authors who received each award<br>• Grant numbers awarded to each author<br>• The full name of each funder<br>• URL of each funder website<br>• Did the sponsors or funders play any role in the study design, data collection and analysis, decision to publish, or preparation of the manuscript?<br>• **NO** - Include this sentence at the end of your statement: *The funders had no role in study design, data collection and analysis, decision to publish, or preparation of the manuscript.*<br>• **YES** - Specify the role(s) played.<br><br>* typeset	M.O. received Grants-in-Aid from the Health, Labour and Welfare Sciences Research Grants, Japan (grant no. H29-Sukoyaka-001).
**Competing Interests**<br><br>Use the instructions below to enter a competing interest statement for this submission. On behalf of all authors,	The authors have declared no competing interests exist.

disclose any competing interests that could be perceived to bias this work—acknowledging all financial support and any other relevant financial or non-financial competing interests.<br><br>This statement **will appear in the published article** if the submission is accepted. Please make sure it is accurate. View published research articles from *PLOS ONE* for specific examples.<br><br>**NO authors have competing interests**<br><br>Enter: *The authors have declared that no competing interests exist.*<br><br>**Authors with competing interests**<br><br>Enter competing interest details beginning with this statement:<br><br>*I have read the journal's policy and the authors of this manuscript have the following competing interests: [insert competing interests here]*<br><br>* typeset	
**Ethics Statement**<br><br>Enter an ethics statement for this submission. This statement is required if the study involved:<br><br>• Human participants<br>• Human specimens or tissue<br>• Vertebrate animals or cephalopods<br>• Vertebrate embryos or tissues<br>• Field research<br><br>Write "N/A" if the submission does not require an ethics statement.<br><br>General guidance is provided below. Consult the submission guidelines for detailed instructions. **Make sure that all information entered here is included in the Methods section of the manuscript.**	Concerning the legal aspect, we have Act on the Protection of Personal Information. But any personal information at postmortem is out of regulation of this law. The ethical committee of the Institutional Review Board for Clinical Research, Tokai University, approved this survey with a referential number of 16R-172, and permitted to gather the anonymized data set to one primary investigator without informed consent, as long as research purpose. But the committee required us to show a sample data set, and to disclose the project on the website as an optout manner (http://forensic.med.u-tokai.ac.jp/ethicscommittee/images/ethicscommittee_02.pdf, in Japanese). This study was also approved by the respective ethical committees of the faculties as a collaborative study. On behalf to reflect editor's concern, statements were also added into the revised manuscript.

Powered by Editorial Manager® and ProduXion Manager® from Aries Systems Corporation

**Format for specific study types**<br><br>**Human Subject Research (involving human participants and/or tissue)**<br>• Give the name of the institutional review board or ethics committee that approved the study<br>• Include the approval number and/or a statement indicating approval of this research<br>• Indicate the form of consent obtained (written/oral) or the reason that consent was not obtained (e.g. the data were analyzed anonymously)<br><br>**Animal Research (involving vertebrate animals, embryos or tissues)**<br>• Provide the name of the Institutional Animal Care and Use Committee (IACUC) or other relevant ethics board that reviewed the study protocol, and indicate whether they approved this research or granted a formal waiver of ethical approval<br>• Include an approval number if one was obtained<br>• If the study involved *non-human primates*, add *additional details* about animal welfare and steps taken to ameliorate suffering<br>• If anesthesia, euthanasia, or any kind of animal sacrifice is part of the study, include briefly which substances and/or methods were applied<br><br>**Field Research**<br><br>Include the following details if this study involves the collection of plant, animal, or other materials from a natural setting:<br>• Field permit number<br>• Name of the institution or relevant body that granted permission	
**Data Availability**<br><br>Authors are required to make all data underlying the findings described fully available, without restriction, and from the time of publication. PLOS allows rare exceptions to address legal and ethical concerns. See the PLOS Data Policy and FAQ for detailed information.	Yes - all data are fully available without restriction

A Data Availability Statement describing where the data can be found is required at submission. Your answers to this question constitute the Data Availability Statement and **will be published in the article**, if accepted.<br><br>**Important:** Stating 'data available on request from the author' is not sufficient. If your data are only available upon request, select 'No' for the first question and explain your exceptional situation in the text box.<br><br>Do the authors confirm that all data underlying the findings described in their manuscript are fully available without restriction?	
**Describe where the data may be found in full sentences. If you are copying our sample text, replace any instances of XXX with the appropriate details.**<br><br>• If the data are **held or will be held in a public repository**, include URLs, accession numbers or DOIs. If this information will only be available after acceptance, indicate this by ticking the box below. For example: *All XXX files are available from the XXX database (accession number(s) XXX, XXX.).*<br>• If the data are all contained **within the manuscript and/or Supporting Information files**, enter the following: *All relevant data are within the manuscript and its Supporting Information files.*<br>• If neither of these applies but you are able to provide **details of access elsewhere**, with or without limitations, please do so. For example:<br><br>*Data cannot be shared publicly because of [XXX]. Data are available from the XXX Institutional Data Access / Ethics Committee (contact via XXX) for researchers who meet the criteria for access to confidential data.*<br><br>*The data underlying the results presented in the study are available*	All relevant data are within the manuscript and its Supporting Information files.

from (include the name of the third party and contact information or URL).<br>• This text is appropriate if the data are owned by a third party and authors do not have permission to share the data.<br><br>* typeset	
Additional data availability information:	

Powered by Editorial Manager® and ProduXion Manager® from Aries Systems Corporation

Manuscript revised Click here to access/download;Manuscript;Manuscript.docx

Circumstances and factors of sudden infancy deaths during sleep in Japan

Motoki Osawa[1*], Yasuhiro Ueno[2], Noriaki Ikeda[3], Kazuya Ikematsu[4], Takuma Yamamoto[5], Wataru Irie[6], Shuji Kozawa[7], Hirokazu Kotani[8], Hideki Hamayasu[8], Takehiko Murase[4], Keita Shingu[4], Marie Sugimoto[2], Ryoko Nagao[1], and Yu Kakimoto[1]

[1] Department of Forensic Medicine, Tokai University School of Medicine, Isehara, Kanagawa, Japan
[2] Department of Legal Medicine, Kobe University Graduate School of Medicine, Kobe, Hyogo, Japan
[3] Department of Forensic Pathology and Sciences, Graduate School of Medical Science, Kyushu University, Fukuoka, Japan
[4] Department of Forensic Pathology and Science, Graduate School of Biomedical Sciences Nagasaki University, Nagasaki, Japan
[5] Department of Legal Medicine, Hyogo College of Medicine, Nishinomiya, Hyogo, Japan
[6] Department of Legal Medicine, Kitasato University School of Medicine, Sagamihara, Kanagawa, Japan
[7] Department of Forensic Medicine and Sciences, Mie University School of Medicine, Tsu, Mie, Japan
[8] Department of Forensic Medicine and Molecular Pathology, Kyoto University Graduate School of Medicine, Kyoto, Japan

[*] Corresponding author

E-mail: osawa@is.icc.u-tokai.ac.jp (MO)

Abstract

Background: Sudden unexpected death in infancy (SUDI) comprises both natural and unnatural causes of death. However, a few epidemiological surveys have investigated SUDI in Japan.

Objective: This retrospective study was conducted to investigate the latest trends of circumstances and risk factors of SUDI cases in which collapse occurred during sleep.

Methods: Forensic pathology sections from eight universities participated in the selection of subjects from 2013 to 2018. Data obtained from the checklist form were analyzed based on information at postmortem.

Results: There were 259 SUDI cases consisting of 145 male infants and 114 female infants with a mean birth weight of 2888 ± 553 and 2750 ± 370 g, respectively. Deaths most frequently occurred among infants at 1 month of age (18%). According to population data as the control, the odds ratio (95% confidence interval) of mother's age ≤19 years was 11.1 (6.9–17.7) compared with ages 30–39. The odds ratio for the fourth- and later born infants was 5.2 (3.4–7.9) compared with the frequency of first-born infants. The most frequent time of day for discovery was between 7 and 8 o'clock, and the time difference from the last seen alive was a mean of 4.1 h. Co-sleeping was recorded for 61%, and the prone position was found for 40% of cases at discovery. Mother's smoking habit exhibited an odds ratio of 4.5 (2.9–5.8).

Conclusion: This study confirmed the trends that have been observed for sudden infant death syndrome; particularly, very high odds ratios were evident for teenage mothers and later birth order in comparison with those in other developed countries. Neglect was suspected in some cases of the prolonged time to discovery of unreactive infants. To our knowledge, this is the first report of an extensive survey of SUDI during sleep in Japan.

Introduction

Sudden infant death syndrome (SIDS) is the possible cause-of-death for sudden infant death during sleep, in which all known identifiable conditions that might engender sudden and unexpected death must be excluded by postmortem examinations. However, several pathologists have changed their diagnostic preferences since 2004 primarily because of the difficult distinction of SIDS from accidental asphyxia or natural diseases such as arrhythmias and metabolic disorders. [1-3] Reluctance to use the term has decreased the number globally over the years. [4-6] In Japan, around 0.5 per 1,000 livebirths (LBs) of annual SIDS rate was recorded in the 1990s), but recent diagnostic numbers have decreased to fewer than 0.1. [7]

Currently, another broad term has become popular, i.e., sudden unexpected death in infancy (SUDI) or sudden unexpected infant death (SUID). Although SUDI/SUID originally has been used as an umbrella term for the initial presentation of explained or unexplained infant deaths, it is interpreted to include several categories such as SIDS (R95), ill-defined and unknown cause of mortality (R99), and ~~accidental suffocation or strangulation in bed (W75).~~ [8] Several attempts have been made to categorize SUDI/SUID, but the terminology and classification have not yet been defined clearly. [9]

In recent years, a protocol of investigation items has been standardized. [10,11] For instance, in the U.S., the Centers for Disease Control and Prevention published guidelines and a reporting form, which is designated as the SUID Investigation Reporting Form. [12] In Japan, a list of items to be investigated is used as a similar checklist form in cases of infant death. [13] A system is in operation for clinicians and pathologists to ascertain circumstances and to investigate background factors.

Information from death scene investigation (DSI) acquired by experts is indispensable to fill out the form. [14] Furthermore, the maternity passbook, which records information about the mother and the child during pregnancy as well as after childbirth, is beneficial. We previously analyzed forensic autopsy cases of sudden infant deaths after vaccination using this passbook. [15]

While the childcare environment differs according to region and time, there are few epidemiological data describing infant deaths during sleep in Japan. [16] Takatsu et al. [17] exhibited the trends of SUID cases from 1982 to 2006. This population-based retrospective study was conducted to investigate the latest trends of sleep-related SUDI cases using the checklist form including the maternity passbook at multiple centers.

Methods

SUDI cases during sleep were recruited from autopsy files for the period of 6 years from 2013 to 2018 to obtain a statistically significant number. Inclusion criteria for cases were age not less than 1 week and not more than 12 months, and the collapse occurring during sleep in an unexpected manner. Based on the cause and manner of death, infant deaths were grouped as follows: (1) infants who died of SIDS, (2) infants who died of other natural diseases, (3) infants who died of accidental injuries, (4) infants who died of non-accidental injuries, and (5) infants with unexplained manner of death. [18] The term ‘unexplained’ implies insufficient evidence of the causative disease or event. [9] In this study, we selected cases of groups (1) and (5) and those of suspected accidental suffocation during sleep even though pathological diagnosis with natural disease of group (2).

Postmortem examinations included histology, toxicology, biochemistry, virology, and bacteriology. [19,20] Tests for assessing inherited metabolic disorders were conducted nationwide in the routine examination for newborns. [21] Genetic testing for arrhythmic disorders was also conducted for cases examined in this study. [22] Data used for analysis consisted of DSI information, therapeutic information in emergency care, and maternity passbook. The checklist form, consisting of 41 items, was filled in initially by each center. Then the lists were transferred to one site to confirm unclear issues and aggregate the data.

The forensic pathology sections of the following eight universities participated in this study: Kitasato University School of Medicine, Mie University School of Medicine, Kyoto University Graduate School of Medicine, Hyogo College of Medicine, Kobe University Graduate School of Medicine, Graduate School of Medical Science Kyushu University, Graduate School of Biomedical Sciences Nagasaki University, and Tokai University School of Medicine. The areas of these facilities cover six prefectures without regional bias in the country. Further, the covered area contained approximately 14% of the entire population resides, and this percentage was applied to ~~calculate~~ the annual rate per 1000. Every sudden infant death had been autopsied, but there could have been some deaths that had not received autopsy outside major centers in Japan, whose exact number remained unknown. Although DSI was performed by the police for all cases, not all items were optimal, particularly, for the sleep environment such as sleep surface, wrapping, and clothing. The principal investigator obtained approval for this retrospective study from the Institutional Review Board for Clinical Research, Tokai University. This study was also approved by the respective ethical committees of the faculties as a collaborative study. All data were fully anonymized before the

analyzing investigators accessed them. The study protocol was disclosed to the public at the website.

The number of 263 cases were originally registered to this project. We checked each candidate case carefully at a meeting, and selected subjects in which terminal events remained at speculation irrespective of the diagnosis in the death certificate. Among them, 4 cases were eliminated from the reasons of not found in sleep environment (n = 3) and expected course of terminal heart failure (n = 1). The original causes of death (n = 259) were SIDS and suspicious SIDS in 94 cases (36%), unexplained in 75 cases (29%), potential asphyxia in bed in 51 cases (20%), and other natural causes in 39 cases (15%). Suspected causes of asphyxia were supposed to be due to accidental overlay and swallowing in bed. Inflammation of the airway, including bronchitis, accounted for 22 cases, comprising the largest group among "others." Pathological findings do not always reflect the terminal event c[illegible]se. Pathologists tend to favor any apparent histological findings as the cause of death whether it was fatal or not. Despite of such histological evidence, the pathologists reconsidered that these cases might also be regarded as sleep-related SUDI because of co-sleeping, in which coexistent factors may have served as contributors in causing death.

In forensic pathology, most of infant deaths are SUDI. ~~Since we had a small number of non-SUDI cases that were suitable for the control, we used~~ ~~national~~ population data as a substitute for the control cases in a comparison with the present data. The statistical data of LBs recorded during 2013–2018 in the Japanese population at the National Institute of Population and Social Security Research (http://www.ipss.go.jp/p-info/) were used as the control. Prevalence information of regular tobacco consumption was available at Japan Tobacco Inc. as a questionnaire survey performed in 2016

(https://www.jti.co.jp/investors/press_releases/2016/0728_01.html). The numbers of smokers and nonsmokers in the 20s and 30s of female volunteers were used for the control.

Logistic regression analyses based on population data were performed to determine the associations of independent variables as estimated using the odds ratio and confidence interval (CI). For significant differences, the *p* value was calculated using chi-square tests. Statistical analyses were conducted using BellCurve in Excel, ver. 3.20 (Social Survey Research Information Co., Tokyo, Japan).

Results

A total of 259 cases were collected at multiple centers for the 6-year period. The circumstances and factors were investigated using the checklist form filled with information at postmortem.

The annual frequency of SUDI during sleep was estimated to approximately 0.31 per 1000 LBs. However, the value could be slightly underestimated because of the possibility that some cases could have been out of management by these facilities.

Table 1 presents the number of subjects according to sex, birth weight, gestation week, maternal age, parity and maternal smoking habit along with the population data. Table 2 summarizes the odds ratios (95% CI) and *p* values in terms of the related factors.

Table 1 Number of SUDI subjects and LBs

Factors	No. (%) of	No. (%) of LBs*	Approx. annual

		SUDI		rate per 1000 LBs
Sex	Male	145 (56%)	3,015,822 (51%)	0.34
	Female	114 (44%)	2,864,653 (49%)	0.28
	Total	259 (100%)	5,880,475 (100%)	0.31
Birth weight:	< 2,500	21 (15%)	252,678 (8%)	0.59
Male	≥ 2,500	116 (85%)	2,762,747 (92%)	0.30
	Total	137 (100%)	3,015,425 (100%)	0.32
Birth weight:	< 2,500	23 (21%)	304,624 (11%)	0.54
Female	≥ 2,500	85 (79%)	2,559,683 (89%)	0.24
	Total	108 (100%)	2,864,307 (100%)	0.27
Gestation week	< 37	31 (13%)	324,829 (6%)	0.68
	≥ 37	203 (87%)	5,546,995 (94%)	0.26
	Total	234 (100%)	5,871,824 (100%)	0.28
Maternal age at	≤ 19	21 (9%)	67,675 (1%)	2.22
delivery (years)	20-24	63 (26%)	500,757 (9%)	0.90
	25-29	58 (24%)	1,538,223 (26%)	0.27
	30-34	53 (22%)	2,124,833 (36%)	0.18
	35-39	43 (17%)	1,335,169 (23%)	0.23
	40-44	7 (3%)	305,543 (5%)	0.16
	≥ 45	0	8,268 (0%)	
	Total	245 (100%)	5,880,468 (100%)	0.30
Parity	1	77 (31%)	2,745,441 (47%)	0.20
	2	93 (38%)	2,145,351 (37%)	0.31
	3	46 (19%)	760,283 (13%)	0.43
	4	20 (8%)	157,536 (3%)	0.91
	≥ 5	10 (4%)	50,823 (1%)	1.41
	Total	246 (100%)	5,859,434 (100%)	0.30
Maternal	Non-smoker	106 (66%)	1,983 (89%)	-
Smoking habit	Smoker	55 (34%)	254 (11%)	-
	Total	161 (100%)	2,237 (100%)	

*: Japanese population data represent the sum of LBs during 2013 to 2018.

Table 2 Odds ratios (95% CI) and *p* values of SUDI cases to population data

	Factors	Odds ratio	*p* value
Sex	Male	1.2 (0.9–1.5)	$p = .13$
	Female (ref. group)	1.0	

Birth weight; Male	< 2,500	2.0 (1.3–3.2)	$p < .01$
	≥ 2,500 (ref. group)	1.0	
Birth weight; Female	< 2,500	2.3 (1.4–3.6)	$p < .001$
	≥ 2,500 (ref. group)	1.0	
Gestation week	< 37	2.6 (1.8–3.8)	$p < .001$
	≥ 37 (ref. group)	1.0	
Maternal age at delivery (years)	≤ 19	11.1 (6.9–17.7)	$p < .001$
	20-29	2.1 (1.6–2.8)	$p < .001$
	30-39 (ref. group)	1.0	
	≥ 40	0.8 (0.4–1.7)	$p = .56$
Parity	1 (ref. group)	1.0	
	2–3	1.7 (1.3–2.3)	$p < .001$
	≥ 4	5.1 (3.4–7.8)	$p < .001$
Maternal smoking habit	Non-smoker (ref. group)	1.0	
	Smoker	4.1 (2.9–5.8)	$p < .001$

Age

Fig 1 shows the age distribution at the time of death. It was observed that deaths most frequently occurred in infants at 1 month of age, consisting of 45 cases (18%). The number was found to decrease with age. Deaths occurring within 6 months after birth accounted for 180 cases (72%).

Fig 1. Age distribution of SUDI infants during sleep (*n* = 259) examined in this study. Key indicates the birth order of infants, in which the column is divided into four groups of the first-born infants (blank), the second- and third-born ones (gray), more than the fourth-born ones (black), and unknown ones (diagonal).

Birth weight and gestation weeks

The mean (± S.D.) birth weight of SUDI infants was 2885 ± 556 g for male subjects (n = 137) and 2763 ± 466 g for female subjects (n = 108). These birth weights were lower by 191 g (6%) and 227 g (8%), respectively, than the national mean birth weights of 3076 g for males and 2990 g for females recorded in 2017. The incidence of low birth weight infants was significantly higher than the control group in both sexes. For low birth weight infants, the odds ratio of over 2.0 was observed in both the male and female groups. Infants of premature birth were found to be 2.6 times more likely to die from SUDI than those of mature birth.

Maternal age and birth order

The odds ratio of incidence of infant death of mothers whose age ≤19 years was the highest at 11.1 compared with mothers aged 30–39 years, and that for mothers aged 20–29 years was 2.1, which showed significant differences. This finding indicated that mothers of a younger age, especially teenage, should be considered as the most important risk factor for the occurrence of SUDI during sleep.

In terms of the birth-order distribution, there were 31% of first-born infants, 38% of second-born infants, 19% of third-born infants, 8% of fourth-born infants, 2% (6 cases) of fifth-born infants, 1% (3 cases) of sixth-born infants, and 0.4% (1 case) of seventh-born infant. The odds ratio to the fatal frequency among the first-born infants clearly indicated that later birth order constituted an important risk factor. Moreover, as shown in Figure 1, there were more first-born infant deaths within 2 months in age (38/92) than those after 3 months (39/154) ($p < 0.001$).

Time of discovery and sleeping position

Fig 2A shows the distribution of the time of day when an unresponsive infant was found. There were 30 cases (12%) found between 7 and 8 o'clock a.m., which was the most frequent time. A large peak was evident between 6 and 9 o'clock in the morning.

Fig 2B depicts that the duration between the last time the infant was found alive and the time of discovery of being unresponsive (n = 222), which varied widely from approximately 10 min to 13 h. The mean duration was 4.1±2.7 h. The collapse was discovered within 6 h in the majority of cases (n = 184, 83%).

Fig 2. Time of discovery. A. Distribution of the time of the day at which the first responder found an unresponsive infant (n = 246); B. the time difference between the time the infant was last seen alive and first found deceased (n = 222).

The first responder (n = 252), who discovered the unresponsive infant, was the mother in 188 cases (75%), followed by the father in 49 cases (19%), a grandmother in 8 cases (3%), a childminder in 2 cases (1%), and others in 6 cases (2%).

Co-sleeping was recorded for 143 cases (61%) among a total of 230 available cases. Table 3 presents the child sleeping position when the collapse was discovered. The prone position in late SUDI infants accounted for 40% of cases, and there were 19% of cases with the prone position even in 0 to 2-month-old subjects who cannot roll over.

Table 3 Sleep position at the scene

Position	Total (0–11 month)	0–2 month
Supine	117 (52%)	60 (72%)
Prone	89 (40%)	16 (19%)
Side	16 (7%)	7 (9%)
Others	2 (1%)	0
Total	224 (100%)	83 (100%)

Maternal smoking habit

The descriptions in the maternity passbook entries are considered to reflect the smoking habits before and during the early phase of pregnancy. We attempted to obtain the smoking rate of the mothers of SUDI cases. A significant risk of SUDI was evident with an odds ratio of 4.5 compared with the general rate. Although there were limited cases wherein the information related to the number of cigarettes ($n = 30$) was available, the mean number was 11 cigarettes/day.

Discussion

Since we had a small number of control cases that were suitable for a case-control study, the national population data was used as a substitute for the reference. In addition, there was a limitation that data were not fully available for each item because of the retrospective approach. The analysis might be accompanied by any ~~potential~~ imprecision, ~~however the~~ high odd ratios were evident in low birth weight, premature birth, teenage mothers, later birth order infants and maternal smoking habit.

These results of the present investigation of sleep-related SUDI cases were consistent with risk factors such the smoking habit of parents in the large epidemiological surveys of SIDS. [23] However, some differences were evident. The peak age of death is generally 2 months in SIDS surveys, [4,24] but that among the present SUDI infants was 1 month of age. We thought that the difference should be caused by that a particularly higher risk was evident among teenage mothers than that found in an earlier study [25], and that more first-born infants were dead during 0–2 months of age.

The most frequent birth order associated with infant death due to SUDI during sleep was the second birth order. Blair et al. [26] reported that SIDS was most frequent among first-born children in the UK, although it was earlier presumed to be frequent in large families. Data from Taiwan indicate that the first-, second-, and third- and later born children account for 36%, 40%, and 24% of SIDS, respectively. [27] The distribution in the present study was more similar to that reported in Taiwan.

Traditional bedding of cotton mat, known as futon, on the floor is common in Japan. Therefore, it is more appropriate to use the term co-sleeping (sharing a sleeping surface) than bed-sharing. Such co-sleeping is a common style of sleep. Tokutake et al. [17] reported that 84% of mothers practice co-sleeping, of whom half also practice breastfeeding. The father was found to be the first responder in up to 20% of cases, and in most of these cases the father also co-slept and discovered the infant death upon awakening. The risk of SIDS among infants who co-sleep was found to be significantly high in several earlier studies. [28,29] Nevertheless, the effects of co-sleeping on the occurrence of SUDI, if any, could not be evaluated in this study because of a variety of co-sleeping styles and the absence of good control subjects.

It is a traditional practice in Japan to lay infants in the supine position. However,

40% of infants were found in the prone position, of which frequency was higher than that reported in an earlier study. [17] Li et al. [30] reported that 60% of SIDS infants were found in the prone position in the United States. It is possible that turning over by infants during sleep is a causal factor. However, approximately 28% of 0 to 2-month-old infants who were unable to turn over were found in the prone and side positions. They might have been placed in the prone position or been breastfed during co-sleeping, but the original position was not recorded sufficiently at DSI.

A striking finding was the prolonged time to discovery. In nine cases, it spent more than 10 hours to find the unreactive infants. No apparent infanticide was involved in all subjects, however neglect by parents was suspected in a couple of cases from circumstances at DSI. We think that the time difference should be an important indicator to suspect careless infant rearing.

A relationship between the occurrence of SIDS and the smoking habit of parents has been found in Japan. [31] In the present investigation, the incidence of pregnant mother’s smoking among SUDI cases was 34%. This incidence in the general female population was reported as 11%, which also resulted in the high odds ratio of 4.5 in this study. According to Anderson et al., [32] the incidence of SUDI more than doubles when a parent is smoking during the period of pregnancy. The odds ratio increases along with the number of cigarettes up to 20. It is evident that infants co-sleeping with someone who smokes exhibit the highest risk for SUDI. [33]

Pasquale-Styles et al. [34] reported that asphyxia and suffocation occur more than presumed in many situations such as bed-sharing, overlay, wedging, prone position, obstruction of the nose and mouth, and coverage of the head. Postmortem findings alone are not generally sufficient to explain the cause of these deaths. Consequently, the

diagnoses often lack consistency. [3,4,7] To avoid preventable any types of SUDI, including SIDS, such various causes of accidental suffocation, and unexplained causes, it is important to identify high risk factors from the study based on the wide variety of cases. [17] In addition, there exists a difficulty of the current situation in Japan, particularly in DSI that is performed by police officers who are not well trained. Garstang et al. [24] indicated that police-led DSI does not comply with practical information. After a new law related to child health was enacted in 2018, child death reviews will be introduced to the society in the near future. These reviews in combination with multiple agencies will be helpful in investigating the sleeping environments of infants in detail.

In conclusion, we conducted an effective epidemiological analysis of sleep-related SUDI using the checklist form. This approach has revealed the present critical features prevailing in the country. This report displayed the latest trends of SUDI in Japan.

Acknowledgements

We thank Tomohiro Nozima for his extensive support like inputting the large data and mailing.

References

1. Krous HF, Beckwith JB, Byard RW, Rognum TO, Bajanowski T, Corey T, et al. Sudden infant death syndrome and unclassified sudden infant deaths: a definitional and diagnostic approach. Pediatrics. 2004;114:234-238.
2. Malloy MH, MacDorman M. Changes in the classification of sudden unexpected infant deaths: United States, 1992-2001. Pediatrics. 2005;115:1247-1253.
3. Camperlengo LT, Shapiro-Mendoza CK, Kim SY. Sudden infant death syndrome: diagnostic practices and investigative policies, 2004. Am J Forensic Med Pathol. 2012;33:197-201.
4. Moon RY. Task Force on Sudden Infant Death Syndrome. SIDS and other sleep related infant deaths: expansion of recommendations for a safe infant sleeping environment. Pediatrics. 2011;128:1030-1039.
5. Fleming PJ, Blair PS, Pease A. Sudden unexpected death in infancy: aetiology, pathophysiology, epidemiology and prevention in 2015. Arch Dis Child. 2015;100:984-988.
6. Matthews TJ, MacDorman MF, Thoma ME. Infant mortality statistics from the 2013 period linked birth/infant death data set. Natl Vital Stat Rep. 2015;64:1-30.
7. Taylor BJ, Garstang J, Engelberts A, Obonai T, Cote A, Freemantle J, et al. International comparison of sudden unexpected death in infancy rates using a newly proposed set of cause-of-death codes. Arch Dis Child. 2015;100:1018-1023.
8. Goldstein RD, Blair PS, Sens MA, Shapiro-Mendoza CK, Krous HF, Rognum TO, et al. Inconsistent classification of unexplained sudden deaths in infants and children hinders surveillance, prevention and research: recommendations from the 3rd International Congress on Sudden Infant and Child Death. Forensic Sci Med

Pathol. 2019;15:622-628.

9. Shapiro-Mendoza CK, Camperlengo L, Ludvigsen R, Cottengim C, Anderson RN, Andrew T, et al. Classification system for the Sudden Unexpected Infant Death Case Registry and its application. Pediatrics. 2014;134:e210-9.

10. Corey TS, Hanzlick R, Howard J, Nelson C, Krous H. A functional approach to sudden unexplained infant deaths. Am J Forensic Med Pathol. 2007;28:271-277.

11. Bennett T, Martin LJ, Heathfield LJ. Global trends in the extent of death scene investigation performed for sudden and unexpected death of infant (SUDI) cases: A systematic review. Forensic Sci Int. 2019;301:435-444.

12. Centers for Disease Control and Prevention. Sudden Unexplained Infant Death Investigation Reporting Form 2007. www.cdc.gov/sids/pdf/suidiform2- 1-2010.pdf. Accessed 1 Nov 2019.

13. Guideline for SIDS diagnosis, ver. 2. Ministry of Health, Labour and Welfare, Japan. (in Japanese), 2012. http://www.mhlw.go.jp/bunya/kodomo/sids_guideline.html of subordinate document. Accessed 2 Nov 2019.

14. Erck Lambert AB, Parks SE, Camperlengo L, Cottengim C, Anderson RL, Covington TM, et al. Death scene investigation and autopsy practices in sudden unexpected infant deaths. J Pediatr. 2016;174:84-90.

15. Osawa M, Nagao R, Kakimoto Y, Kakiuchi Y, Satoh F. Sudden infant death after vaccination: Survey of forensic autopsy files. Am J Forensic Med Pathol. 2019;40:232-237.

16. Takatsu A, Shigeta A, Sakai K, Abe S. Risk factors, diagnosis and prevention of sudden unexpected infant death. Leg Med. 2007;9:76-82.

17. Tokutake C, Haga A, Sakaguchi K, Samejima A, Yoneyama M, Yokokawa Y, et al. Infant suffocation incidents related to co-sleeping or breastfeeding in the side-lying position in Japan. Tohoku J Exp Med. 2018; 246:121-130.
18. Matthews T, McDonnell M, McGarvey C, Loftus G, O'Regan M. A multivariate "time based" analysis of SIDS risk factors. Arch Dis Child. 2004;89:267-271.
19. Howatson AG. The autopsy for sudden unexpected death in infancy. Curr Diagn Pathol. 2006;12:173-183.
20. Kakimoto Y, Seto Y, Ochiai E, Satoh F, Osawa M. Cytokine elevation in sudden death with respiratory syncytial virus: A Case Report of 2 Children. Pediatrics. 2016;138:e20161293.
21. Yamamoto T, Mishima H, Mizukami H, Fukahori Y, Umehara T, Murase T, et al. Metabolic autopsy with next generation sequencing in sudden unexpected death in infancy: Postmortem diagnosis of fatty acid oxidation disorders. Mol Genet Metab Rep. 2015;5:26-32.
22. Osawa M, Inaoka Y, Ochiai E, Hasegawa I, Satoh F. Variable single C repetitive tract in KCNQ1: Postmortem molecular testing in a sudden and unexpected death. Exp Clin Cardiol. 2014;20:927-930.
23. Moon RY, Hauck FR. Risk Factors and Theories In: Duncan JR, Byard RW, editors. SIDS Sudden Infant and Early Childhood Death: The Past, the Present and the Future. Adelaide (AU): University of Adelaide Press; 2018.
24. Garstang J, Ellis C, Sidebotham P. An evidence-based guide to the investigation of sudden unexpected death in infancy. Forensic Sci Med Pathol. 2015;11:345-357.
25. Colson ER, Willinger M, Rybin D, Heeren T, Smith LA, Lister G, et al. Trends and factors associated with infant bed sharing, 1993-2010: the National Infant Sleep

Position Study. JAMA Pediatr. 2013;167:1032-1037.
26. Blair PS, Sidebotham P, Berry PJ, Evans M, Fleming PJ. Major epidemiological changes in sudden infant death syndrome: a 20-year population-based study in the UK. *Lancet*. 2006;367:314-319.
27. Chang HP, Li CY, Chang YH, Hwang SL, Su YH, Chen CW. Sociodemographic and meteorological correlates of sudden infant death in Taiwan. Pediatr Int. 2013;55:11-16.
28. Hauck FR, Herman SM, Donovan M, Iyasu S, Merrick Moore C, Donoghue E, et al. Sleep environment and the risk of sudden infant death syndrome in an urban population: the Chicago Infant Mortality Study. Pediatrics. 2003;111:1207-1214.
29. Blair PS, Sidebotham P, Pease A, Fleming PJ. Bed-sharing in the absence of hazardous circumstances: is there a risk of sudden infant death syndrome? An analysis from two case-control studies conducted in the UK. PLoS One. 2014; 9:e107799.
30. Li L, Fowler D, Liu L, Ripple MG, Lambros Z, Smialek JE. Investigation of sudden infant deaths in the State of Maryland (1990-2000). Forensic Sci Int. 2005; 148:85-92.
31. Tanaka T, Kato N. Evaluation of child care practice factors that affect the occurrence of sudden infant death syndrome: Interview conducted by public health nurses. Environ Health Prev Med. 2001;6:117-120.
32. Anderson TM, Lavista Ferres JM, Ren SY, Moon RY, Goldstein RD, Ramirez JM, et al. Maternal smoking before and during pregnancy and the risk of sudden unexpected infant death. Pediatrics. 2019;143:e20183325.
33. James C, Klenka H, Manning D. Sudden infant death syndrome: bed sharing with

mothers who smoke. Arch Dis Child. 2003;88:112-113.

34. Pasquale-Styles MA, Tackitt PL, Schmidt CJ. Infant death scene investigation and the assessment of potential risk factors for asphyxia: a review of 209 sudden unexpected infant deaths. J Forensic Sci. 2007;52:924-929.

Figure 1

Click here to access/download;Figure;Figure1-3ADCfinal.pptx

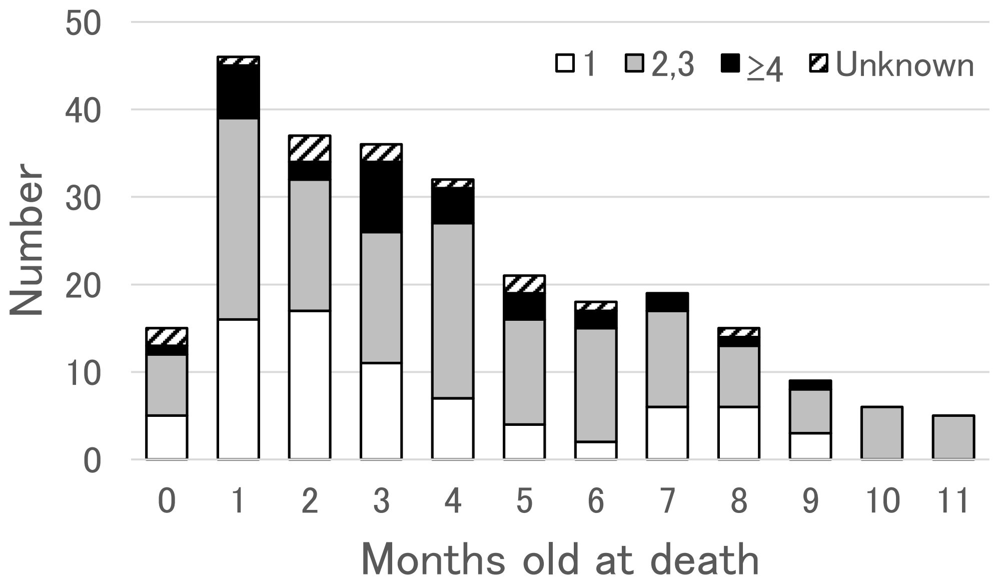

Figure 2

Click here to access/download;Figure;Figure2-2ADCfinal.pptx

A

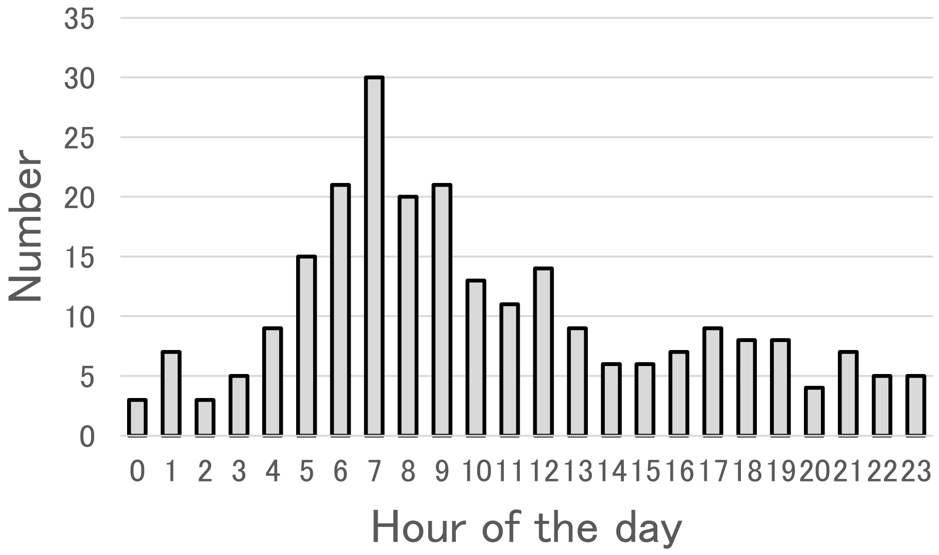

B

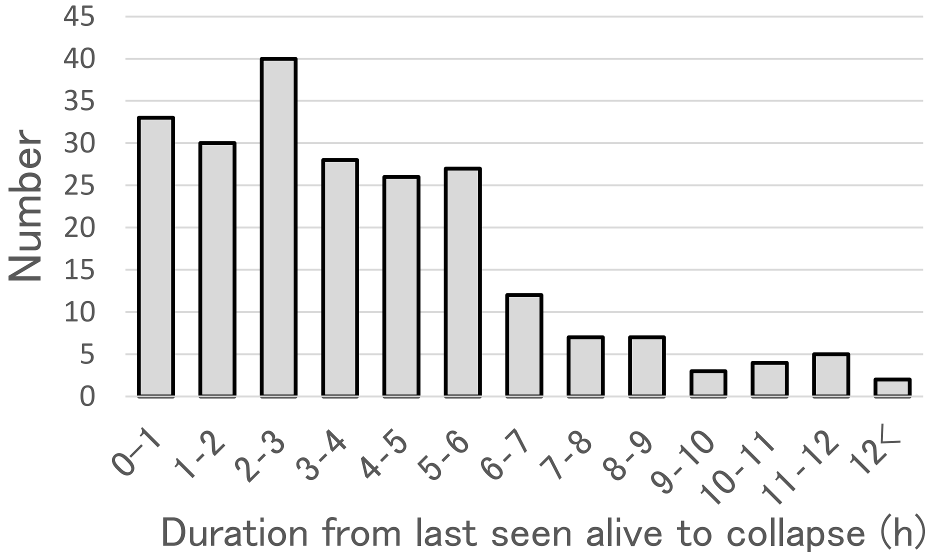

Click here to access/download
Supporting Information
all data sheet.xlsx

Circumstances and factors of ~~sleep-related~~ sudden infancy deaths in Japan

Commented [大澤　資樹1]: sudden infancy deaths during sleep

Motoki Osawa[1*], Yasuhiro Ueno[2], Noriaki Ikeda[3], Kazuya Ikematsu[4], Takuma Yamamoto[5], Wataru Irie[6], Shuji Kozawa[7], Hirokazu Kotani[8], Hideki Hamayasu[8], Takehiko Murase[4], Keita Shingu[4], Marie Sugimoto[2], Ryoko Nagao[1], and Yu Kakimoto[1]

[1] Department of Forensic Medicine, Tokai University School of Medicine, Isehara, Kanagawa, Japan

[2] Department of Legal Medicine, Kobe University Graduate School of Medicine, Kobe, Hyogo, Japan

[3] Department of Forensic Pathology and Sciences, Graduate School of Medical Science, Kyushu University, Fukuoka, Japan

[4] Department of Forensic Pathology and Science, Graduate School of Biomedical Sciences Nagasaki University, Nagasaki, Japan

[5] Department of Legal Medicine, Hyogo College of Medicine, Nishinomiya, Hyogo, Japan

[6] Department of Legal Medicine, Kitasato University School of Medicine, Sagamihara, Kanagawa, Japan

[7] Department of Forensic Medicine and Sciences, Mie University School of Medicine, Tsu, Mie, Japan

[8] Department of Forensic Medicine and Molecular Pathology, Kyoto University Graduate School of Medicine, Kyoto, Japan

[*] Corresponding author

E-mail: osawa@is.icc.u-tokai.ac.jp (MO)

Abstract

Background: Sudden unexpected death in infancy (SUDI) comprises both natural and unnatural causes of death. However, a few epidemiological surveys have investigated SUDI in Japan.

Objective: This retrospective study was conducted to investigate the ~~recent~~ trends of circumstances and risk factors of ~~sleep-related~~ SUDI cases.

Methods: Forensic pathology sections from eight universities participated in the selection of subjects from 2013 to 2018. Data obtained from the checklist form were analyzed based on information at postmortem.

Results: There were 259 SUDI cases consisting of 145 male infants and 114 female infants with a mean birth weight of 2888 ± 553 and 2750 ± 370 g, respectively. Deaths most frequently occurred among infants at 1 month of age (18%). According to ~~population-based analyses~~, the odds ratio (95% confidence interval) of mother's age ≤19 years was 11.1 (6.9–17.7) compared with ages 30–39. The odds ratio for the fourth- and later born infants was 5.2 (3.4–7.9) compared with the frequency of first-born infants. The most frequent time of day for discovery was between 7 and 8 o'clock. Co-sleeping was recorded for 61%, and the prone position was found for 40% of cases at discovery. Mother's smoking habit exhibited an odds ratio of 4.5 (2.9–5.8).

Conclusion: This study confirmed the trends that have been observed for sudden infant death syndrome; particularly, very high odds ratios were evident for teenage mothers and later birth order in comparison with those in other developed countries. ~~The child of a young mother tended to die within 2 months of age.~~ To our knowledge, this is the first report of an extensive survey of ~~sleep-related~~ SUDI in Japan.

Commented [O2]: latest

Commented [大澤　資樹3]: SUDI cases in which collapse occurred during sleep

Commented [大澤　資樹4]: population data as the control

Commented [O5]: o'clock, and the time difference from the last seen alive was a mean of 4.1 h.

Commented [O6]: Neglect was suspected in some cases of the prolonged time to discovery of unreactive infants.

Commented [大澤　資樹7]: SUDI during sleep

Introduction

Sudden infant death syndrome (SIDS) is the possible cause-of-death for sudden infant death during sleep, in which all known identifiable conditions that might engender sudden and unexpected death must be excluded by postmortem examinations. However, several pathologists have changed their diagnostic preferences since 2004 primarily because of the difficult distinction of SIDS from accidental asphyxia or natural diseases such as arrhythmias and metabolic disorders. [1-3] Reluctance to use the term has decreased the number globally over the years. [4-6] In Japan, around ~~500 cases of SIDS were~~ recorded in the 1990s, but recent diagnostic numbers have decreased to fewer than ~~100 cases per year~~. [7]

Currently, another broad term has become popular, i.e., sudden unexpected death in infancy (SUDI) or sudden unexpected infant death (SUID). Although SUDI/SUID originally has been used as an umbrella term for the initial presentation of explained or unexplained infant deaths, it is interpreted to include several categories such as SIDS (R95), ill-defined and unknown cause of mortality (R99), and accidental suffocation or strangulation in bed (W75). [8] Several attempts have been made to categorize SUDI/SUID, but the terminology and classification have not yet been defined clearly. [9]

In recent years, a protocol of investigation items has been ~~officially~~ standardized. [10,11] For instance, in the U.S., the Centers for Disease Control and Prevention published guidelines and a reporting form, which is designated as the SUID Investigation Reporting Form. [12] In Japan, a list of items to be investigated is used as a checklist form in cases of infant death. [13] A system is in operation for clinicians and pathologists to ascertain circumstances and to investigate background factors.

Commented [大澤　資樹8]: 0.5 per 1,000 livebirths (LBs) of annual SIDS rate was

Commented [大澤　資樹9]: 0.1

Commented [大澤　資樹10]: deleted

Commented [大澤　資樹11]: a similar checklist

Information from death scene investigation (DSI) acquired by experts is indispensable to fill out the form. [14] Furthermore, the maternity passbook, which records information about the mother and the child during pregnancy as well as after childbirth, is beneficial. We previously analyzed forensic autopsy cases of sudden infant deaths after vaccination using this passbook. [15]

While the childcare environment differs according to region and time, there are few epidemiological data describing infant deaths during sleep in Japan. ~~[16,17]~~ This population-based retrospective study was conducted to investigate the ~~recent~~ trends of sleep-related SUDI using the checklist form at multiple centers.

Methods

~~Deaths of sleep-related SUDI cases were extracted~~ from autopsy files for the period of 6 years from 2013 to 2018. Inclusion criteria for cases were age not more than 12 months, and the collapse occurring during sleep in an unexpected manner. Based on the cause and manner of death, infant deaths were grouped as follows: (1) infants who died of SIDS, (2) infants who died of other natural diseases, (3) infants who died of accidental injuries, (4) infants who died of non-accidental injuries, and (5) infants with unexplained manner of death. [18] The term ‘unexplained’ implies insufficient evidence of the causative disease or event. [9] In this study, we selected cases of groups (1) and (5) and those of suspected accidental suffocation during sleep.

Postmortem examinations included histology, toxicology, biochemistry, virology, and bacteriology. [19,20] Tests for assessing inherited metabolic disorders were conducted nationwide in the routine examination for newborns. [21] Genetic testing for

Commented [大澤　資樹12]: [16] Takatsu et al. [17] exhibited the trends of SUID cases from 1982 to 2006.

Commented [大澤　資樹13]: latest

Commented [大澤　資樹14]: SUDI cases using the checklist form including the maternity passbook

Commented [O15]: SUDI cases during sleep were recruited

Commented [大澤　資樹16]: from 2013 to 2018 to obtain a statistically significant number

Commented [大澤　資樹17]: age not less than 1 week and not more than 12 months

Commented [大澤　資樹18]: during sleep even though pathological diagnosis with natural disease of group (2)

arrhythmic disorders was also conducted for cases examined in this study. [22] Data used for analysis consisted of DSI information, therapeutic information in emergency care, and maternity passbook. The checklist form, consisting of 41 items, was filled in initially by each center. Then the lists were transferred to one site to confirm unclear issues and aggregate the data.

The forensic pathology sections of the following eight universities participated in this study: Kitasato University School of Medicine, Mie University School of Medicine, Kyoto University Graduate School of Medicine, Hyogo College of Medicine, Kobe University Graduate School of Medicine, Graduate School of Medical Science Kyushu University, Graduate School of Biomedical Sciences Nagasaki University, and Tokai University School of Medicine. The areas of these facilities cover six prefectures ~~in which~~ approximately 14% of the entire population resides, and this percentage was applied to calculate the annual rate per 1000. Every sudden infant death had been autopsied, but there could have been some deaths that had not received autopsy outside major centers in Japan, whose exact number remained unknown. Although DSI was performed by the police for all cases, not all items were optimal, particularly, for the sleep environment such as sleep surface, wrapping, and clothing. The principal investigator obtained approval for this retrospective study from the Institutional Review Board for Clinical Research, Tokai University. This study was also approved by the respective ethical committees of the faculties as a collaborative study.

The original causes of death (*n* = 259) were SIDS and suspicious SIDS in 94 cases (36%), unexplained in 75 cases (29%), potential asphyxia in bed in 51 cases (20%), and other causes in 39 cases (15%). ~~We investigated each candidate case carefully at a meeting, and selected subjects in which terminal events remained at speculation~~

Commented [O19]: without regional bias in the country. Further, the covered area contained

Commented [大澤　資樹20]: study. All data were fully anonymized before the analyzing investigators accessed them. The study protocol was disclosed to the public at the website.

Commented [大澤　資樹21]: The number of 263 cases were originally registered to this project. We checked each candidate case carefully at a meeting, and selected subjects in which terminal events remained at speculation irrespective of the diagnosis in the death certificate. Among them, 4 cases were eliminated from the reasons of not found in sleep environment (n = 3) and expected course of terminal heart failure (n = 1). The …

Commented [大澤　資樹22]: other natural causes

~~irrespective of the diagnosis in the death certificate.~~ Suspected causes of asphyxia were supposed to be due to accidental overlay and swallowing in bed. Inflammation of the airway, including bronchitis, accounted for 22 cases, comprising the largest group among “others.” Despite of such histological evidence, the pathologists reconsidered that these cases might also be regarded as sleep-related SUDI because of co-sleeping, in which coexistent factors may have served as contributors in causing death.

The statistical data of ~~live births~~ recorded during 2013–2018 in the Japanese population at the National Institute of Population and Social Security Research (http://www.ipss.go.jp/p-info/) were used as the control. Prevalence information of regular tobacco consumption was available at Japan Tobacco Inc. as a questionnaire survey performed in 2016 (https://www.jti.co.jp/investors/press_releases/2016/0728_01.html). The numbers of smokers and nonsmokers in the 20s and 30s of female volunteers were used for the control.

Logistic regression analyses based on population data were performed to determine the associations of independent variables as estimated using the odds ratio and confidence interval (CI). For significant differences, the p value was calculated using chi-square tests. Statistical analyses were conducted using BellCurve in Excel, ver. 3.20 (Social Survey Research Information Co., Tokyo, Japan).

Results

A total of 259 cases were collected at multiple centers for the 6-year period. The circumstances and factors were investigated using the checklist form filled with

Commented [大澤　資樹23]: deleted

Commented [大澤　資樹24]: Pathological findings do not always reflect the clinical stage of disease. Pathologists tend to favor any apparent histological findings as the cause of death whether it was fatal or not. Despite …

Commented [大澤　資樹25]: In forensic pathology, most of infant deaths are SUDI. Since we had a small number of non-SUDI cases that were suitable for the control, we used national population data as a substitute for the control cases in a comparison with the present data. The …

Commented [大澤　資樹26]: LBs

information at postmortem.

The annual frequency of ~~sleep-related SUDI~~ was estimated to approximately 0.3 per 1000 ~~births~~. However, the value could be slightly underestimated because of the possibility that some cases could have been out of management by these facilities.

Table 1 presents the number of subjects according to sex, birth weight, gestation week, maternal age, parity and maternal smoking habit along with the population data. Table 2 summarizes the odds ratios (95% CI) and p values in terms of the related factors. ~~The factors were also analyzed for two groups, i.e., the early group in which death occurred within 2 months of age (n = 98) and the late group in which death occurred after 3 months (n = 161).~~

Table 1 Number of ~~infants of SUID subjects and live births~~

Factors	No. of ~~SUID~~	~~No. of early/late~~	No. of ~~live births~~*	Approx. annual rate per ~~1000~~
Sex				
Male	145 (56%)	~~53/92~~	3,015,822	0.34
Female	114 (44%)	~~45/69~~	2,864,653	0.28
Total	259 (100%)	~~98/161~~	5,880,475	0.31
Birth weight				
Male				
< 2,500	21 (15%)	~~3/48~~	252,678	0.59
≥ 2,500	116 (85%)	~~18/68~~	2,762,747	0.30
Total	137 (100%)	~~21/116~~	3,015,425	0.32
Female				
< 2,500	23 (21%)	~~9/14~~	304,624	0.54
≥ 2,500	85 (79%)	~~36/49~~	2,559,683	0.24
Total	108 (100%)	~~45/63~~	2,864,307	0.27
Gestation week				
< 37	31 (13%)	~~8/23~~	324,829	0.68
≥ 37	203 (87%)	~~84/119~~	5,546,995	0.26

Commented [大澤　資樹27]: SUDI during sleep

Commented [大澤　資樹28]: LBs

Commented [大澤　資樹29]: deleted

Commented [大澤　資樹30]: SUDI subjects and LBs

Commented [大澤　資樹31]: SUDI

Commented [大澤　資樹32]: The column is deleted.

Commented [大澤　資樹33]: LBs

Commented [大澤　資樹34]: 1000 LBs

Total	234 (100%)	~~92/142~~	5,871,824	0.28
Maternal age at delivery (years)				
≤ 19	21 (9%)	~~12/9~~	67,675	2.22
20-24	63 (26%)	~~18/45~~	500,757	0.90
25-29	58 (24%)	~~26/32~~	1,538,223	0.27
30-34	53 (22%)	~~15/38~~	2,124,833	0.18
35-39	43 (17%)	~~19/24~~	1,335,169	0.23
40-44	7 (3%)	~~3/4~~	305,543	0.16
≥ 45	0		8,268	
Total	245 (100%)	~~93/152~~	5,880,468	0.30
Parity				
1	77 (31%)	~~38/39~~	2,745,441	0.20
2	93 (38%)	~~26/67~~	2,145,351	0.31
3	46 (19%)	~~20/26~~	760,283	0.43
4	20 (8%)	~~5/15~~	157,536	0.91
≥ 5	10 (4%)	~~4/6~~	50,823	1.41
Total	246 (100%)	~~93/153~~	5,859,434	0.30
Maternal Smoking habit				
Non-smoker	106 (66%)	~~44/62~~	1,983	-
Smoker	55 (34%)	~~14/41~~	254	-
Total	161 (100%)	~~58/103~~	2,237	

*: Japanese population data represent the sum of ~~live births~~ during 2013 to 2018.

Commented [大澤　資樹35]: LBs

Table 2 Odds ratios (95% CI) and *p* values of ~~SUID including early and late occurrences~~

Commented [大澤　資樹36]: SUDI cases to population data

Factors	Total (0–11 month)	~~Early (0–2 month)~~	~~Late (3–11 month)~~
Sex			
Male	1.2 (0.9–1.5), *p* = 0.13	~~1.1 (0.8–1.7), *p* = 0.58~~	~~1.3 (0.9–1.7), *p* = 0.14~~
Female (ref. group)	1.0	~~1.0~~	~~1.0~~
Birth weight			
Male			
< 2,500	2.0 (1.3–3.2), *p* = 0.003	~~0.7 (0.2–2.2), *p* = 0.52~~	~~2.9 (1.7–4.9), *p* < 0.001~~
≥ 2,500 (ref. group)	1.0	~~1.0~~	~~1.0~~
Female			
< 2,500	2.3 (1.4–3.6), *p* < 0.001	~~2.1 (1.0–4.4), *p* = 0.042~~	~~2.4 (1.3–4.3), *p* = 0.003~~

Commented [大澤　資樹37]: These two columns are deleted.

≥ 2,500 (ref. group)	1.0	~~1.0~~	~~1.0~~
Gestation week			
< 37	2.6 (1.8–3.8), $p < 0.001$	~~1.6 (0.8–3.4), $p = 0.18$~~	~~3.3 (2.1–5.2), $p < 0.001$~~
≥ 37 (ref. group)	1.0	~~1.0~~	~~1.0~~
Maternal age at delivery (years)			
≤ 19	11.1 (6.9–17.7), $p < 0.001$	~~18.0 (9.3–34.8), $p < 0.001$~~	~~7.4 (3.7–14.9), $p < 0.001$~~
20-29	2.1 (1.6–2.8), $p < 0.001$	~~2.2 (1.4–3.5), $p < 0.001$~~	~~2.1 (1.5–2.9), $p < 0.001$~~
30-39 (ref. group)	1.0	~~1.0~~	~~1.0~~
≥ 40	0.8 (0.4–1.7), $p = 0.56$	~~1.0 (0.3–3.2), $p = 0.96$~~	~~0.7 (0.3–2.0), $p = 0.51$~~
Parity			
1 (ref. group)	1.0	~~1.0~~	~~1.0~~
2–3	1.7 (1.3–2.3), $p < 0.001$	~~1.1 (0.7–1.7), $p = 0.61$~~	~~2.3 (1.6–3.3), $p < 0.001$~~
≥ 4	5.1 (3.4–7.8), $p < 0.001$	~~3.1 (1.5–6.5), $p = 0.0012$=~~	~~7.1 (4.2–12.1), $p < 0.001$~~
Maternal smoking habit			
Non-smoker (ref. group)	1.0	~~1.0~~	~~1.0~~
Smoker	4.1 (2.9–5.8), $p < 0.001$	~~2.5 (1.3–4.6), $p = 0.002$~~	~~5.2 (3.4–7.8), $p < 0.001$~~

Age

Fig 1 shows the age distribution at the time of death. It was observed that deaths most frequently occurred in infants at 1 month of age, consisting of 45 cases (18%). The number was found to decrease with age. Deaths occurring within 6 months after birth accounted for 180 cases (72%).

Fig 1. Age distribution of ~~sleep-related~~ SUDI infants (*n* = 259) examined in this study. Key indicates the birth order of infants, in which the column is divided into four groups of the first-born infants (blank), the second- and third-born ones (gray), more than the fourth-born ones (black), and unknown ones (diagonal).

Commented [大澤　資樹38]: SUDI infants during sleep

Birth weight and gestation weeks

The mean (± S.D.) birth weight of SUDI infants was 2885 ± 556 g for male subjects and 2763 ± 466 g for female subjects. These birth weights were lower by 191 g (6%) and 227 g (8%), respectively, than the national mean birth weights of 3076 g for males and 2990 g for females recorded in 2017. The ~~percentage~~ of low birth weight infants was significantly higher than the control group in both sexes. For low birth weight infants, ~~the highest odds ratio of 2.9 was observed in the late male group.~~

Infants of premature birth were found to be 2.6 times more likely to die from SUDI than those of mature birth. ~~The late group also showed higher odds ratio than the early group.~~

Maternal age and birth order

The odds ratio of incidence of infant death of mothers whose age ≤19 years was the highest at 11.1 compared with mothers aged 30–39 years, and that for mothers aged 20–29 years was 2.1, which showed significant differences. ~~Furthermore, the child of a teenage mother tended to die within 2 months of age, compared with other generations (12/21 to 81/225, p = 0.04).~~ This finding indicated that mothers of a younger age, especially teenage, should be considered as the most important risk factor for the occurrence of ~~sleep-related~~ SUDI.

In terms of the birth-order distribution, there were 31% of first-born infants, 38% of second-born infants, 19% of third-born infants, 8% of fourth-born infants, 2% (6 cases) of fifth-born infants, 1% (3 cases) of sixth-born infants, and 0.4% (1 case) of seventh-born infant. The odds ratio to the fatal frequency among the first-born infants clearly indicated that later birth order constituted an important risk factor. Moreover, as shown in Figure 1, there were more first-born ~~infants in the early group (38/92) than in~~

Commented [大澤　資樹39]: subjects (n = 137)

Commented [大澤　資樹40]: subjects (n = 108)

Commented [大澤　資樹41]: incidence

Commented [大澤　資樹42]: the odds ratio of over 2.0 was observed in both the male and female groups

Commented [大澤　資樹43]: This sentence is deleted.

Commented [O44]: Thus sebtence is deleted.

Commented [大澤　資樹45]: SUDI during sleep

~~the late group (39/154)~~ ($p < 0.001$).

Time of discovery and sleeping position

Fig 2A shows the distribution of the time of day when an unresponsive infant was found. There were 30 cases (12%) found between 7 and 8 o'clock a.m., which was the most frequent time. A large peak was evident between 6 and 9 o'clock in the morning.

Fig 2B depicts that the duration between the last time the infant was found alive and the time of discovery of being unresponsive (n = 222), which varied widely from approximately 10 min to 13 h. The mean duration was 4.1±2.7 h. The collapse was discovered within 6 h in the majority of cases (n = 184, 83%).

Fig 2. Time of discovery. A. Distribution of the time of the day at which the first responder found an unresponsive infant (n = 246); B. the time difference between the time the infant was last seen alive and first found deceased (n = 222).

The first responder (n = 252), who discovered the unresponsive infant, was the mother in 188 cases (75%), followed by the father in 49 cases (19%), a grandmother in 8 cases (3%), a childminder in 2 cases (1%), and others in 6 cases (2%).

Co-sleeping was recorded for 143 cases (61%) among a total of 230 available cases. Table 3 presents the child sleeping position when the collapse was discovered. The prone position in late SUDI infants accounted for 40% of cases, and there were 19% of cases with the prone position ~~in the early group~~.

Table 3 Sleep position at the scene

Commented [大澤　資樹46]: infant deaths within 2 months in age (38/92) than those after 3 months (39/154)

Commented [大澤　資樹47]: even in 0 to 2-month-old subjects who cannot roll over

	Total (0–11 month)	Early (0–2 month)	Late (3–11 month)
Spine	117 (52%)	60 (72%)	57 (40%)
Prone	89 (40%)	16 (19%)	73 (52%)
Side	16 (7%)	7 (9%)	9 (6%)
Others	2 (1%)	0	2 (1%)
Total	224 (100%)	83 (100%)	141 (100%)

Maternal smoking habit

The descriptions in the maternity passbook entries are considered to reflect the smoking habits before and during the early phase of pregnancy. We attempted to obtain the smoking rate of the mothers of SUDI cases. A significant risk of SUDI was evident with an odds ratio of 4.5 compared with the general rate. Although there were limited cases wherein the information related to the number of cigarettes (n = 30) was available, the mean number was 11 cigarettes/day.

Discussion

Results of the present investigation of sleep-related SUDI cases were consistent with risk factors such the smoking habit of parents in the large epidemiological surveys of SIDS. [23] However, some differences were evident. The peak age of death is generally 2 months in SIDS surveys, [4,24] but that among the present SUDI infants was 1 month of age. A particularly higher risk was observed among teenage mothers than that found in an earlier study. [25] Moreover, the mothers tended to lose their infants at age 0–2

Commented [O48]: Position

Commented [大澤　資樹49]: 0–2 month

Commented [大澤　資樹50]: This column is deleted.

Commented [大澤　資樹51]: Supine

Commented [O52]: Since we had a small number of control cases that were suitable for a case-control study, the national population data was used as a substitute for the reference. In addition, there was a limitation that data were not fully available for each item because of the retrospective approach. The analysis might be accompanied by any potential imprecision, however the high odd ratios were evident in low birth weight, premature birth, teenage mothers, later birth order infants and maternal smoking habit.
These results

~~months.~~

The most frequent birth order associated with infant death due to ~~sleep-related~~ SUDI was the second birth order. Blair et al. [26] reported that SIDS was most frequent among first-born children in the UK, although it was earlier presumed to be frequent in large families. Data from Taiwan indicate that the first-, second-, and third- and later born children account for 36%, 40%, and 24% of SIDS, respectively. [27] The distribution in the present study was more similar to that reported in Taiwan.

Traditional bedding of cotton mat, known as futon, on the floor is common in Japan. Therefore, it is more appropriate to use the term co-sleeping (sharing a sleeping surface) than bed-sharing. Co-sleeping is a common style of sleep~~, and~~ Tokutake et al. [17] reported that 84% of mothers practice co-sleeping, of whom half also practice breastfeeding. The father was found to be the first responder in up to 20% of cases, and in most of these cases the father also co-slept and discovered the infant death upon awakening. The risk of SIDS among infants who co-sleep was found to be significantly high in several earlier studies. [28,29] Nevertheless, the effects of co-sleeping on the occurrence of SUDI, if any, could not be evaluated in this study because of the absence of good control subjects.

It is a traditional practice in Japan to lay infants in the supine position. However, 40% of infants were found in the prone position, of which frequency was higher than that reported in an earlier study. [17] Li et al. [30] reported that 60% of SIDS infants were found in the prone position in the United States. It is possible that turning over by infants during sleep is a causal factor. However, approximately 28% of ~~infants in the early group~~ who were unable to turn over were found in the prone and side positions. They might have been placed in the prone position or been breastfed during co-sleeping,

Commented [O53]: We thought that the difference should be caused by that a particularly higher risk was evident among teenage mothers than that found in an earlier study [25], and that more first-born infants were dead during 0–2 months of age.

Commented [大澤　資樹54]: SUDI during sleep

Commented [O55]: Such co-sleeping is a common style of sleep. Tokutake …
(Separated into two sentences)

Commented [O56]: a variety of co-sleeping styles and the absence

Commented [大澤　資樹57]: 0 to 2-month-old infants

but the original position was not recorded sufficiently at DSI.

A relationship between the occurrence of SIDS and the smoking habit of parents has been found in Japan. [31] In the present investigation, the incidence of pregnant mother's smoking among SUDI cases was 34%. This incidence in the general female population was reported as 11%, which also resulted in the high odds ratio of 4.5 in this study. According to Anderson et al., [32] the incidence of SUDI more than doubles when a parent is smoking during the period of pregnancy. The odds ratio increases along with the number of cigarettes up to 20. It is evident that infants co-sleeping with someone who smokes exhibit the highest risk for ~~SUID~~. [33]

Pasquale-Styles et al. [34] reported that asphyxia and suffocation occur more than presumed in many situations such as bed-sharing, overlay, wedging, prone position, obstruction of the nose and mouth, and coverage of the head. Postmortem findings alone are not generally sufficient to explain the cause of these deaths. Consequently, the diagnoses often lack consistency. [3,4,7] In addition, there exists a difficulty of the current situation in Japan, particularly in DSI that is performed by police officers who are not well trained. Garstang et al. [24] indicated that police-led DSI does not comply with practical information. After a new law related to child health was enacted in 2018, child death reviews will be introduced to the society in the near future. These reviews in combination with multiple agencies will be helpful in investigating the sleeping environments of infants in detail.

In conclusion, we conducted an effective epidemiological analysis of sleep-related SUDI using the ~~official~~ checklist form. This approach has revealed the present critical features prevailing in the country. This report ~~is the first of an extensive study~~ of SUDI in Japan.

Commented [大澤　資樹58]: A striking finding was the prolonged time to discovery. In nine cases, it spent more than 10 hours to find the unreactive infants. No apparent infanticide was involved in all subjects, however neglect by parents was suspected in a couple of cases from circumstances at DSI. We think that the time difference should be an important indicator to suspect careless infant rearing.
A relationship …

Commented [大澤　資樹59]: SUDI

Commented [大澤　資樹60]: To avoid preventable any types of SUDI, including SIDS, such various causes of accidental suffocation, and unexplained causes, it is important to identify high risk factors from the study based on the wide variety of cases. [17] In addition, …

Commented [大澤　資樹61]: deleted

Commented [大澤　資樹62]: displayed the latest trends

Acknowledgements

We thank Tomohiro Nozima for his extensive support like inputting the large data and mailing.

References

1. Krous HF, Beckwith JB, Byard RW, Rognum TO, Bajanowski T, Corey T, et al. Sudden infant death syndrome and unclassified sudden infant deaths: a definitional and diagnostic approach. Pediatrics. 2004;114:234-238.
2. Malloy MH, MacDorman M. Changes in the classification of sudden unexpected infant deaths: United States, 1992-2001. Pediatrics. 2005;115:1247-1253.
3. Camperlengo LT, Shapiro-Mendoza CK, Kim SY. Sudden infant death syndrome: diagnostic practices and investigative policies, 2004. Am J Forensic Med Pathol. 2012;33:197-201.
4. Moon RY. Task Force on Sudden Infant Death Syndrome. SIDS and other sleep related infant deaths: expansion of recommendations for a safe infant sleeping environment. Pediatrics. 2011;128:1030-1039.
5. Fleming PJ, Blair PS, Pease A. Sudden unexpected death in infancy: aetiology, pathophysiology, epidemiology and prevention in 2015. Arch Dis Child. 2015;100:984-988.
6. Matthews TJ, MacDorman MF, Thoma ME. Infant mortality statistics from the 2013 period linked birth/infant death data set. Natl Vital Stat Rep. 2015;64:1-30.
7. Taylor BJ, Garstang J, Engelberts A, Obonai T, Cote A, Freemantle J, et al. International comparison of sudden unexpected death in infancy rates using a newly proposed set of cause-of-death codes. Arch Dis Child. 2015;100:1018-1023.
8. Goldstein RD, Blair PS, Sens MA, Shapiro-Mendoza CK, Krous HF, Rognum TO, et al. Inconsistent classification of unexplained sudden deaths in infants and children hinders surveillance, prevention and research: recommendations from the 3rd International Congress on Sudden Infant and Child Death. Forensic Sci Med

Pathol. 2019;15:622-628.

9. Shapiro-Mendoza CK, Camperlengo L, Ludvigsen R, Cottengim C, Anderson RN, Andrew T, et al. Classification system for the Sudden Unexpected Infant Death Case Registry and its application. Pediatrics. 2014;134:e210-9.
10. Corey TS, Hanzlick R, Howard J, Nelson C, Krous H. A functional approach to sudden unexplained infant deaths. Am J Forensic Med Pathol. 2007;28:271-277.
11. Bennett T, Martin LJ, Heathfield LJ. Global trends in the extent of death scene investigation performed for sudden and unexpected death of infant (SUDI) cases: A systematic review. Forensic Sci Int. 2019;301:435-444.
12. Centers for Disease Control and Prevention. Sudden Unexplained Infant Death Investigation Reporting Form 2007. www.cdc.gov/sids/pdf/suidiform2- 1-2010.pdf. Accessed 1 Nov 2019.
13. Guideline for SIDS diagnosis, ver. 2. Ministry of Health, Labour and Welfare, Japan. (in Japanese), 2012. http://www.mhlw.go.jp/bunya/kodomo/sids_guideline.html of subordinate document. Accessed 2 Nov 2019.
14. Erck Lambert AB, Parks SE, Camperlengo L, Cottengim C, Anderson RL, Covington TM, et al. Death scene investigation and autopsy practices in sudden unexpected infant deaths. J Pediatr. 2016;174:84-90.
15. Osawa M, Nagao R, Kakimoto Y, Kakiuchi Y, Satoh F. Sudden infant death after vaccination: Survey of forensic autopsy files. Am J Forensic Med Pathol. 2019;40:232-237.
16. Takatsu A, Shigeta A, Sakai K, Abe S. Risk factors, diagnosis and prevention of sudden unexpected infant death. Leg Med. 2007;9:76-82.

17. Tokutake C, Haga A, Sakaguchi K, Samejima A, Yoneyama M, Yokokawa Y, et al. Infant suffocation incidents related to co-sleeping or breastfeeding in the side-lying position in Japan. Tohoku J Exp Med. 2018; 246:121-130.
18. Matthews T, McDonnell M, McGarvey C, Loftus G, O'Regan M. A multivariate "time based" analysis of SIDS risk factors. Arch Dis Child. 2004;89:267-271.
19. Howatson AG. The autopsy for sudden unexpected death in infancy. Curr Diagn Pathol. 2006;12:173-183.
20. Kakimoto Y, Seto Y, Ochiai E, Satoh F, Osawa M. Cytokine elevation in sudden death with respiratory syncytial virus: A Case Report of 2 Children. Pediatrics. 2016;138:e20161293.
21. Yamamoto T, Mishima H, Mizukami H, Fukahori Y, Umehara T, Murase T, et al. Metabolic autopsy with next generation sequencing in sudden unexpected death in infancy: Postmortem diagnosis of fatty acid oxidation disorders. Mol Genet Metab Rep. 2015;5:26-32.
22. Osawa M, Inaoka Y, Ochiai E, Hasegawa I, Satoh F. Variable single C repetitive tract in KCNQ1: Postmortem molecular testing in a sudden and unexpected death. Exp Clin Cardiol. 2014;20:927-930.
23. Moon RY, Hauck FR. Risk Factors and Theories In: Duncan JR, Byard RW, editors. SIDS Sudden Infant and Early Childhood Death: The Past, the Present and the Future. Adelaide (AU): University of Adelaide Press; 2018.
24. Garstang J, Ellis C, Sidebotham P. An evidence-based guide to the investigation of sudden unexpected death in infancy. Forensic Sci Med Pathol. 2015;11:345-357.
25. Colson ER, Willinger M, Rybin D, Heeren T, Smith LA, Lister G, et al. Trends and factors associated with infant bed sharing, 1993-2010: the National Infant Sleep

Position Study. JAMA Pediatr. 2013;167:1032-1037.
26. Blair PS, Sidebotham P, Berry PJ, Evans M, Fleming PJ. Major epidemiological changes in sudden infant death syndrome: a 20-year population-based study in the UK. *Lancet*. 2006;367:314-319.
27. Chang HP, Li CY, Chang YH, Hwang SL, Su YH, Chen CW. Sociodemographic and meteorological correlates of sudden infant death in Taiwan. Pediatr Int. 2013;55:11-16.
28. Hauck FR, Herman SM, Donovan M, Iyasu S, Merrick Moore C, Donoghue E, et al. Sleep environment and the risk of sudden infant death syndrome in an urban population: the Chicago Infant Mortality Study. Pediatrics. 2003;111:1207-1214.
29. Blair PS, Sidebotham P, Pease A, Fleming PJ. Bed-sharing in the absence of hazardous circumstances: is there a risk of sudden infant death syndrome? An analysis from two case-control studies conducted in the UK. PLoS One. 2014; 9:e107799.
30. Li L, Fowler D, Liu L, Ripple MG, Lambros Z, Smialek JE. Investigation of sudden infant deaths in the State of Maryland (1990-2000). Forensic Sci Int. 2005; 148:85-92.
31. Tanaka T, Kato N. Evaluation of child care practice factors that affect the occurrence of sudden infant death syndrome: Interview conducted by public health nurses. Environ Health Prev Med. 2001;6:117-120.
32. Anderson TM, Lavista Ferres JM, Ren SY, Moon RY, Goldstein RD, Ramirez JM, et al. Maternal smoking before and during pregnancy and the risk of sudden unexpected infant death. Pediatrics. 2019;143:e20183325.
33. James C, Klenka H, Manning D. Sudden infant death syndrome: bed sharing with

mothers who smoke. Arch Dis Child. 2003;88:112-113.

34. Pasquale-Styles MA, Tackitt PL, Schmidt CJ. Infant death scene investigation and the assessment of potential risk factors for asphyxia: a review of 209 sudden unexpected infant deaths. J Forensic Sci. 2007;52:924-929.

Reviewer #1:

The authors appreciate the detailed reviewing. According to the raised comments, the manuscript has been rewritten to a revised version. Alterations are indicated as track changes in the revised manuscript. The answers to the raised comments are as follows,

This paper describes the recent status of sudden unexpected death in infancy (SUDI) using the data obtained by a postmortem check in Japan. The study design was a retrospective case-control method. The authors analyzed 259 SUDI cases during 2013-2018 registered in 8 different universities. They found that SUDI most frequently occurred among infants at 1 month of age and significant associations with the infants born to mothers at their teenage, later born infants, and a smoking habit of a mother. They also mentioned possible associations with co-sleeping and prone positioning during sleep. Thus, the study is very important, however, the results from this study are weakened due to the lack of appropriate control subjects. Furthermore, the eligibility and selection criteria of sleep-related SUDI infants among all SUDI infants were not clear in the manuscript.

Answer;
The presented survey is not a typical case-control study. For forensic pathologists, most of the encountered infant deaths are SUDI, and we have only a small number of non-SUDI cases that were suitable for the control. Therefore, in this series of study, we used national population data as a substitute for a comparison with the SUDI cases. Concerning our limitation. a statement was added into the Methods and Discussion sections (lines 130 and 235 of the tack changed text). Please also see lines 142-144 of page 6 and lines 244-245 of page 12 in the revised version.

Major comments.
1 Clarify the relationship between SUDI and sleep-related SUDI in Abstract and Manuscript.

Answer;
In this report, the authors used the term of 'sleep-related SUDI' as SUDI of which collapse was found during sleep. As the reviewer pointed out, the term of 'sleep-related' may mislead readers to sleep inducible sudden death. In the revised version, the term of 'sleep-related' has been replaced by 'during sleep' as possible. This statement was altered in

lines 33 and 50 of the Abstract in the track changed text. Moreover, we changed the title, and other five sites in lines 88, 149, 170, 193 and 242.

2 Method line 89. Explain why the study period of 2013-2018 was selected.

Answer;
In the initial attempt, we selected the five-year-period from 2013 to 2017. However, the number of gathered cases was around 200, which seemed insufficient for statistical analysis. Then, we extended the period one more year to 2018 because we thought that more than 250 cases should be desirable for statistically significant analysis. A phrase was added into the sentence of Methods.

3 Method lone 90-96 and 120-129. Explain the selection and classification methods of the study subjects precisely. Not clear even how many SUDI were registered totally. When and who decided? How to get a final agreement among investigators? Any audit by the third party? This is the most important point of this study.

Answer;
As the reviewer pointed out, the explanation was short in the manuscript. The principle investigator of the first author asked each head to extract SUDI cases during sleep. The number of 263 cases were originally registered to this project. The principle investigator selected them according to the criteria with the five researchers of the collaborating faculties in one room of the Tokai University School of Medicine. We excluded 4 cases of collapses during awakening (n = 3) and excepted death from heart anomaly at terminal stage (n = 1). The steps are described with new sentences in line 120 of the track changed text.

4 Method line 127. Is this stipulation feasible? Need more explanation.

Answer;
Pathological findings do not always reflect the real clinical stage of disease. This is the limitation in pathological diagnosis. For instance, bronchitis or bronchopneumonia was observed under microscopy. If it were severe, he or she might be in bed at hospital. But

the reality was that the baby laid in bed with their parent at home as usual. Pathologists tend to choose any apparent histological findings as the cause of death even though it might be fatal or not. We could not rule out retrospectively the possibility of overlay during sleep. No one have any means to show the evidence of overlay. Two sentences are added in line 127.

5 Method line 111 and 130-132. If this study covered only 14% of the total population in Japan, it would be hard to say “population based” (line 40). Rather say “regional investigation”.

Answer;
It was impossible for us to expand the research to the whole nation of which population is 125 million. This activity was supported by a national grant, but a small amount of budget such as 2,000 US$ was annually available. As the reviewer pointed out, our data of such limited area might not be applicable for the whole population data. But, as mentioned earlier, good control cases were not available for us. We could not find out another strategy except for using population data. Sentences are added to express our situations in more details in lines 130 and 235 of the track changed text.

6 Method line 130-132. It would be better to use the population background of the prefectures where 8 universities belong to.

Answer;
The area to which the eight joined faculties covered metropolitan cities like Fukuoka and Kyoto to rural side like small islands and isolated mountain side. The distribution was biased around the country, we thought that the data should represent the whole nation. Concerning the limitation of regional bias, a sentence is added into line 110 of the track changed text.

7 Results Table 2 line 162-163. Were these odds ratios adjusted against the background distributions of the control?

Answer;

No, the odds ratios were not adjusted. The reviewer should imply the Pearson's χ^2 test, which is calculated based on deviation of each fraction. The mean value and deviation are available for the whole national population data. But it was impossible to obtain the mean and deviation for grouped fractions separately such as < 2500 g and > 2500 g. Further, the χ^2 value of exact method is usually compatible with that in the Pearson's test.

8 Results the same as above. Explain why the early group consists of 0-2 months of age.

Answer;
As the reviewer pointed out, it should be unreasonable to separate the subjects into two groups of 0-2 month-old infants and others. The authors quitted the grouping. Tables 1 and 2 have been revised completely. Further, several sentences concerning this grouping were deleted from the test completely. Please see lines 155-157, 181, 183-184 and 189-191 of the original text.

9 Discussion. Summarize key results without repeating the data already mentioned in Results.

Answer;
The authors added major findings of this study into the first paragraph of the Discussion (line 235 of the track changed text). Please also see lines 246-251 in the revised version.

10 Discussion. Better to make a paragraph of limitations of the study with potential bias or imprecision.

Answer;
As the reviewer pointed out, a paragraph including the statement concerning the limitation is inserted into the beginning of the Discussion, which is the same way as indicated to the last comment.

11 Discussion line 250-252. Be careful about this interpretation because of the lack of control subjects.

Answer;

This sentence is not our interpretation. This is the results that Tokutake et al. investigated [17]. To circumvent the misleading, the sentence is changed a bit.

Minor comments

1 Discussion lines 279 and 286. The authors said the DSI was not reliable while the checklist was an official, very confusing description.

Answer;

The statement was inappropriate. The checklist form is widely utilized among health care stuffs of pediatricians and pathologists. The form is also distributed to police officers in some degree, but it is not mandatory for them to fill in. The authors discarded the word of ‘official’ at two sites. Please see lines 70 and 286 in the original version.

2 Better to show a flow diagram of subjects.

Answer;

The number of originally registered cases was 263. As mentioned earlier, only 4 cases were eliminated from the reasons of not found in sleep environment (n = 3) and fully explained clinical cause of heart disease (n = 1) among them. As mentioned earlier, the statement was inserted into the revised text. No flow diagram has been constructed because of the small number of selection.

Reviewer #2::

The detailed review of our article is appreciated. According to the raised comments, the manuscript has been rewritten to a revised version. Alterations are indicated as track changes in the revised manuscript. The answers to the raised comments are as follows,

Line 61. it might be easier to make a international comparison by giving rates instead of numbers per year, e.g.if rates of SIDS are given as 0.15 per 1000 livebirths

Answer;
As the reviewer advised, the number has changed to the annual rate per 1000 livebirths.

Line 73. Good that Japan has a SIDS investigation form - is it used for by the death scene investigation team or forensic pathologists? The authors might want to mention that it is similar to CDC SUDI investigation form since it is in Japanese.

Answer;
The checklist form is widely utilized among medical stuffs including pediatricians and pathologists. Items in the form is similar to the CDC one. The sentence is changed a bit.

Line 79. It would be interesting as an introduction to mention incidence of SUDI in Japan (0.4(?) per 1000 livebirths from Taylor et al's study) and any findings of sleep related SUDI from previous studies such as ref 17 or from using the DSI form, since that is the authors' major focus, rather than a mention of vaccination related SUDI

Answer;
As the reviewer found, the incidence of 'SUDI' in Japan is around 0.4 per 1000 livebirths, which is shown in the Tayler's article [7]. But what the authors would like to say in this sentence was the number of 'SIDS', not 'SUDI'. In the 1990s, the diagnosis of 'SIDS' was popular, but the diagnosis is considerably avoided among pathologists these days. Anyhow, the aim of our study was not clearly described in the last paragraph of Introduction. One sentence is added into the paragraph on line 82 of the track changed text). Further, the maternity passbook that was used in the last study was very useful for

the present study as well, particularly for the maternal smoking habit. The term of ‘maternity passbook’ is added into the last sentence as well (line 84).

Line 88. The authors' case definition included infants 0-365 days. According to most definitions, unexpected deaths in infants under 7 days of age are excluded from the SUDI category, and instead have been termed “sudden unexpected early neonatal death (SUEND)”. It would be interesting to know how many infants in the study were actually below 7 days of age to see if it would impact the findings related to early or late SUDI.

Answer;
In this revision, we provided the whole data set that was used in this series of studies. The earliest death was a 9-day-old infant. A phrase of ‘age less than 1 week’ is added into line 89 of the track changed text.

Line 134 - alignment of words (for editor)

Answer;
An equal line-width makes the prolonged arrangement. Sorry for no other expression.

Line 157, do the authors mean the later group is in where death occurred at or after 3 months of age .. . What about the infants of 2 months plus to 3 months of age if the later group is AFTER 3 months of age and Early group is within 2 months of age (<= 2 months old). Do the authors mean "below 3 months of age" ?
From the existing literature, the age groups are usually neonatal SUDI (i.e. below 28 days , approximately 1 month of age) and after the neonatal period. Is it an empirical decision or to take into account the preterm babies ?

Answer;
As the reviewer pointed out, it should be unreasonable to separate the subjects into two groups of 0-2 and 3-11-month-old infants. The authors agree with spoiling away the grouping. Tables 1 and 2 have been reconstructed completely. For Table 3, we demonstrated the data within 2-month-old infants because some of SUDI cases found in prone position prior to rolling over, which should be a good finding, we thought.

Line 159 Table 1. "SUDI" is used in the text but "SUID" is used in the Table, suggest to use one term only for consistency. Suggest to add 'livebirths' to become "Approx annual (incidence) rate per 1000 livebirths(LB) "

Answer;
We appreciate finding such careless spell miss. The term has been corrected at a total of three sites. Please see Table 1.

Line 179 - Is it the annual incidence rate of SUDI in low birth weight infants being significantly higher rather than the "percentage", the latter which is not shown in table 1

Answer;
The term of ‘percentage’ is corrected to ‘incidence’, as indicated by the reviewer.

Line 223 Table 3 - typo Supine

Answer;
The spell miss is corrected.

Statistical analysis :
Please excuse me if my understanding of the analysis is incorrect ...
Table 1 -
1.the annual rate per 1000 LB for male infants would be [145*1000/no. of LB (in the 6 years)] divided by 6 = 0.008 and so forth for the other variables. It may also not be necessary to have an annual incidence but just an overall incidence over the 6 years since the annual rate is very low

Answer;
The number was further divided by 0.14, of which number was from the rate of covered population in this study.
145 *1000 / no. of LB (in the 6 years) / 0.14 = 0.34

2. Line 190 and 191 - I am not sure if the number of early SUDI in one age group compared to early SUDI in the rest of the population can be used in chi squared calculation against total SUDI population- should it instead be compared with non event population in the same age group ?

Answer;
The authors agreed with the comment by the reviewer. The distribution of occurrence is not even in the other generations, meaning that the comparison was very rough. As mentioned earlier, we quitted grouping of 0-2 and 3-11 month-old infants. We would like to delete this sentence.

Line 255. The effects of co-sleeping could not be evaluated but perhaps more details if available could be described under findings? Was there any related to wedging, inadvertent suffocation especially in co-sleeping cases ? Or to mention Line 280 ..."not well trained in documentation".

Answer;
Situations of co-sleeping varied among cases such as with mother, with parents and with brothers and sisters, and in the same mattress and the separate mattresses, etc. Actually, two fathers confessed overlay during drunken sleeping. However, the detailed information was not obtained for all. It was difficult to evaluate the effects of co-sleeping further. In the next time, we would like to do another prospective study to evaluate effects of co-sleeping. The sentence has been changed a bit on line 256.

Discussion - Some comment re missing data would be helpful , 14 cases missing in gender , 25 cases in gestation and more than half cases had missing data on maternal smoking - how it may affect the reliability of the data esp with regards to smoking , although this is a known risk factor in sleep related SUDI. Sleeping position was missing in 35 cases. These are understandable given the retrospective nature of the study - so a comment may be useful to the reader

Answer;

Because this was a retrospective study, it was impossible to get all data without any lucks. A statement concerning this limitation was added into the revised text. The sentence was inserted into the first sentence of the first paragraph of Discussion (line 235 in the track changed text). Please also see lines 244-249 in the revised version.

Line 238 - Could the authors comment if the peak age of death at one month is related to the age group being studied to be 0-12 months of age as compared to other SUDI studies where the study group is from 2 to 12 months ?

Answer;
Good point! Other SUDI studies [4,24] showed the peak age to 2 months. As far as we read the articles, no apparent differences were found. However, we thought that the difference in peak age might be caused by that a particularly higher risk was evident among teenage mothers, and that more first-born infants were dead during 0–2 months of age. This message is replaced on lines 239-241.

Prevention issues - of the risk groups are not discussed much . Any comments on how the babies of later birth order might be at risk ?

Answer;
The authors are sorry for no idea about prevention issues to infants in later birth order, because we are not clinicians who care a number of alive patients. But according to the advice from the reviewer, we added a general sentence concerning prevention into line 278 of the track changed text.

Is there a concern about neglect if the duration of an infant seen alive is more than 5-6 hours for those found in the morning, and more than 2-3 hours for those found in the afternoon (since the parents or childminder would be awake then)? Is there a likely delay in reporting due to possible infanticide/ negligence in those reporting that last seen alive was 8 hours ? Or is it a non carer who was reporting his duration? Lack of this data could be a discussion point in your study re improvement in investigation by the social workers or police.
Issues related to DSI itself could be a discussion point on quality improvement in

documentation and investigative process, were there any cases which could have been missed infanticide ?

Answer;

The authors absolutely agree with this comment by the reviewer. A striking finding was the prolonged time of discovery. We thought no apparent infanticide was involved in because DSI was performed by police officers, and pathologists have experiences to find out enforced events. But neglect was highly suspected for a couple of cases, particularly for which more than 10 hours was spent to find the unresponsive infant. A new paragraph was created in the Discussion section (line 266 of the track changed text). Please also see lines 281-285 in the revised version. Moreover, a sentence concerning this finding is added into the last part of Abstract (lines 48-49 in the track changed text).
