# Supplementary material for: Circumstances and factors of sleep-related sudden infancy deaths in Japan
Source: PLoS One. 2020 Aug 21;15(8):e0233253. doi: 10.1371/journal.pone.0233253 (PMC7444554; doi:10.1371/journal.pone.0233253)
Supplement: Attachment — Submitted filename: Responce to Reviwers.docx [file pone.0233253.s002.docx]

Reviewer #1:

The authors appreciate the detailed reviewing. According to the raised comments, the manuscript has been rewritten to a revised version. Alterations are indicated as track changes in the revised manuscript. The answers to the raised comments are as follows,

This paper describes the recent status of sudden unexpected death in infancy (SUDI) using the data obtained by a postmortem check in Japan. The study design was a retrospective case-control method. The authors analyzed 259 SUDI cases during 2013-2018 registered in 8 different universities. They found that SUDI most frequently occurred among infants at 1 month of age and significant associations with the infants born to mothers at their teenage, later born infants, and a smoking habit of a mother. They also mentioned possible associations with co-sleeping and prone positioning during sleep. Thus, the study is very important, however, the results from this study are weakened due to the lack of appropriate control subjects. Furthermore, the eligibility and selection criteria of sleep-related SUDI infants among all SUDI infants were not clear in the manuscript.

Answer;

The presented survey is not a typical case-control study. For forensic pathologists, most of the encountered infant deaths are SUDI, and we have only a small number of non-SUDI cases that were suitable for the control. Therefore, in this series of study, we used national population data as a substitute for a comparison with the SUDI cases. Concerning our limitation. a statement was added into the Methods and Discussion sections (lines 130 and 235 of the tack changed text). Please also see lines 142-144 of page 6 and lines 244-245 of page 12 in the revised version.

Major comments.

1 Clarify the relationship between SUDI and sleep-related SUDI in Abstract and Manuscript.

Answer;

In this report, the authors used the term of ‘sleep-related SUDI’ as SUDI of which collapse was found during sleep. As the reviewer pointed out, the term of ‘sleep-related’ may mislead readers to sleep inducible sudden death. In the revised version, the term of ‘sleep-related’ has been replaced by ‘during sleep’ as possible. This statement was altered in lines 33 and 50 of the Abstract in the track changed text. Moreover, we changed the title, and other five sites in lines 88, 149, 170, 193 and 242.

2 Method line 89. Explain why the study period of 2013-2018 was selected.

Answer;

In the initial attempt, we selected the five-year-period from 2013 to 2017. However, the number of gathered cases was around 200, which seemed insufficient for statistical analysis. Then, we extended the period one more year to 2018 because we thought that more than 250 cases should be desirable for statistically significant analysis. A phrase was added into the sentence of Methods.

3 Method lone 90-96 and 120-129. Explain the selection and classification methods of the study subjects precisely. Not clear even how many SUDI were registered totally. When and who decided? How to get a final agreement among investigators? Any audit by the third party? This is the most important point of this study.

Answer;

As the reviewer pointed out, the explanation was short in the manuscript. The principle investigator of the first author asked each head to extract SUDI cases during sleep. The number of 263 cases were originally registered to this project. The principle investigator selected them according to the criteria with the five researchers of the collaborating faculties in one room of the Tokai University School of Medicine. We excluded 4 cases of collapses during awakening (n = 3) and excepted death from heart anomaly at terminal stage (n = 1). The steps are described with new sentences in line 120 of the track changed text.

4 Method line 127. Is this stipulation feasible? Need more explanation.

Answer;

Pathological findings do not always reflect the real clinical stage of disease. This is the limitation in pathological diagnosis. For instance, bronchitis or bronchopneumonia was observed under microscopy. If it were severe, he or she might be in bed at hospital. But the reality was that the baby laid in bed with their parent at home as usual. Pathologists tend to choose any apparent histological findings as the cause of death even though it might be fatal or not. We could not rule out retrospectively the possibility of overlay during sleep. No one have any means to show the evidence of overlay. Two sentences are added in line 127.

5 Method line 111 and 130-132. If this study covered only 14% of the total population in Japan, it would be hard to say “population based” (line 40). Rather say “regional investigation”.

Answer;

It was impossible for us to expand the research to the whole nation of which population is 125 million. This activity was supported by a national grant, but a small amount of budget such as 2,000 US$ was annually available. As the reviewer pointed out, our data of such limited area might not be applicable for the whole population data. But, as mentioned earlier, good control cases were not available for us. We could not find out another strategy except for using population data. Sentences are added to express our situations in more details in lines 130 and 235 of the track changed text.

6 Method line 130-132. It would be better to use the population background of the prefectures where 8 universities belong to.

Answer;

The area to which the eight joined faculties covered metropolitan cities like Fukuoka and Kyoto to rural side like small islands and isolated mountain side. The distribution was biased around the country, we thought that the data should represent the whole nation. Concerning the limitation of regional bias, a sentence is added into line 110 of the track changed text.

7 Results Table 2 line 162-163. Were these odds ratios adjusted against the background distributions of the control?

Answer;

No, the odds ratios were not adjusted. The reviewer should imply the Pearson’s χ^2^ test, which is calculated based on deviation of each fraction. The mean value and deviation are available for the whole national population data. But it was impossible to obtain the mean and deviation for grouped fractions separately such as < 2500 g and > 2500 g. Further, the χ^2^ value of exact method is usually compatible with that in the Pearson’s test.

8 Results the same as above. Explain why the early group consists of 0-2 months of age.

Answer;

As the reviewer pointed out, it should be unreasonable to separate the subjects into two groups of 0-2 month-old infants and others. The authors quitted the grouping. Tables 1 and 2 have been revised completely. Further, several sentences concerning this grouping were deleted from the test completely. Please see lines 155-157, 181, 183-184 and 189-191 of the original text.

9 Discussion. Summarize key results without repeating the data already mentioned in Results.

Answer;

The authors added major findings of this study into the first paragraph of the Discussion (line 235 of the track changed text). Please also see lines 246-251 in the revised version.

10 Discussion. Better to make a paragraph of limitations of the study with potential bias or imprecision.

Answer;

As the reviewer pointed out, a paragraph including the statement concerning the limitation is inserted into the beginning of the Discussion, which is the same way as indicated to the last comment.

11 Discussion line 250-252. Be careful about this interpretation because of the lack of control subjects.

Answer;

This sentence is not our interpretation. This is the results that Tokutake et al. investigated [17]. To circumvent the misleading, the sentence is changed a bit.

Minor comments

1 Discussion lines 279 and 286. The authors said the DSI was not reliable while the checklist was an official, very confusing description.

Answer;

The statement was inappropriate. The checklist form is widely utilized among health care stuffs of pediatricians and pathologists. The form is also distributed to police officers in some degree, but it is not mandatory for them to fill in. The authors discarded the word of ‘official’ at two sites. Please see lines 70 and 286 in the original version.

2 Better to show a flow diagram of subjects.

Answer;

The number of originally registered cases was 263. As mentioned earlier, only 4 cases were eliminated from the reasons of not found in sleep environment (n = 3) and fully explained clinical cause of heart disease (n = 1) among them. As mentioned earlier, the statement was inserted into the revised text. No flow diagram has been constructed because of the small number of selection.

Reviewer #2::

The detailed review of our article is appreciated. According to the raised comments, the manuscript has been rewritten to a revised version. Alterations are indicated as track changes in the revised manuscript. The answers to the raised comments are as follows,

Line 61. it might be easier to make a international comparison by giving rates instead of numbers per year, e.g.if rates of SIDS are given as 0.15 per 1000 livebirths

Answer;

As the reviewer advised, the number has changed to the annual rate per 1000 livebirths.

Line 73. Good that Japan has a SIDS investigation form - is it used for by the death scene investigation team or forensic pathologists? The authors might want to mention that it is similar to CDC SUDI investigation form since it is in Japanese.

Answer;

The checklist form is widely utilized among medical stuffs including pediatricians and pathologists. Items in the form is similar to the CDC one. The sentence is changed a bit.

Line 79. It would be interesting as an introduction to mention incidence of SUDI in Japan (0.4(?) per 1000 livebirths from Taylor et al's study) and any findings of sleep related SUDI from previous studies such as ref 17 or from using the DSI form, since that is the authors' major focus, rather than a mention of vaccination related SUDI

Answer;

As the reviewer found, the incidence of ‘SUDI’ in Japan is around 0.4 per 1000 livebirths, which is shown in the Tayler’s article [7]. But what the authors would like to say in this sentence was the number of ‘SIDS’, not ‘SUDI’. In the 1990s, the diagnosis of ‘SIDS’ was popular, but the diagnosis is considerably avoided among pathologists these days. Anyhow, the aim of our study was not clearly described in the last paragraph of Introduction. One sentence is added into the paragraph on line 82 of the track changed text). Further, the maternity passbook that was used in the last study was very useful for the present study as well, particularly for the maternal smoking habit. The term of ‘maternity passbook’ is added into the last sentence as well (line 84).

Line 88. The authors' case definition included infants 0-365 days. According to most definitions, unexpected deaths in infants under 7 days of age are excluded from the SUDI category, and instead have been termed “sudden unexpected early neonatal death (SUEND)”. It would be interesting to know how many infants in the study were actually below 7 days of age to see if it would impact the findings related to early or late SUDI.

Answer;

In this revision, we provided the whole data set that was used in this series of studies. The earliest death was a 9-day-old infant. A phrase of ‘age less than 1 week’ is added into line 89 of the track changed text.

Line 134 - alignment of words (for editor)

Answer;

An equal line-width makes the prolonged arrangement. Sorry for no other expression.

Line 157, do the authors mean the later group is in where death occurred at or after 3 months of age .. . What about the infants of 2 months plus to 3 months of age if the later group is AFTER 3 months of age and Early group is within 2 months of age (<= 2 months old). Do the authors mean "below 3 months of age" ?

From the existing literature, the age groups are usually neonatal SUDI (i.e. below 28 days , approximately 1 month of age) and after the neonatal period. Is it an empirical decision or to take into account the preterm babies ?

Answer;

As the reviewer pointed out, it should be unreasonable to separate the subjects into two groups of 0-2 and 3-11-month-old infants. The authors agree with spoiling away the grouping. Tables 1 and 2 have been reconstructed completely. For Table 3, we demonstrated the data within 2-month-old infants because some of SUDI cases found in prone position prior to rolling over, which should be a good finding, we thought.

Line 159 Table 1. "SUDI" is used in the text but "SUID" is used in the Table, suggest to use one term only for consistency. Suggest to add 'livebirths' to become "Approx annual (incidence) rate per 1000 livebirths(LB) "

Answer;

We appreciate finding such careless spell miss. The term has been corrected at a total of three sites. Please see Table 1.

Line 179 - Is it the annual incidence rate of SUDI in low birth weight infants being significantly higher rather than the "percentage", the latter which is not shown in table 1

Answer;

The term of ‘percentage’ is corrected to ‘incidence’, as indicated by the reviewer.

Line 223 Table 3 - typo Supine

Answer;

The spell miss is corrected.

Statistical analysis :

Please excuse me if my understanding of the analysis is incorrect ...

Table 1 -

1.the annual rate per 1000 LB for male infants would be [145*1000/no. of LB (in the 6 years) ] divided by 6 = 0.008 and so forth for the other variables. It may also not be necessary to have an annual incidence but just an overall incidence over the 6 years since the annual rate is very low

Answer;

The number was further divided by 0.14, of which number was from the rate of covered population in this study.

145 *1000 / no. of LB (in the 6 years) / 0.14 = 0.34

2. Line 190 and 191 - I am not sure if the number of early SUDI in one age group compared to early SUDI in the rest of the population can be used in chi squared calculation against total SUDI population- should it instead be compared with non event population in the same age group ?

Answer;

The authors agreed with the comment by the reviewer. The distribution of occurrence is not even in the other generations, meaning that the comparison was very rough. As mentioned earlier, we quitted grouping of 0-2 and 3-11 month-old infants. We would like to delete this sentence.

Line 255. The effects of co-sleeping could not be evaluated but perhaps more details if available could be described under findings? Was there any related to wedging, inadvertent suffocation especially in co-sleeping cases ? Or to mention Line 280 ..."not well trained in documentation".

Answer;

Situations of co-sleeping varied among cases such as with mother, with parents and with brothers and sisters, and in the same mattress and the separate mattresses, etc. Actually, two fathers confessed overlay during drunken sleeping. However, the detailed information was not obtained for all. It was difficult to evaluate the effects of co-sleeping further. In the next time, we would like to do another prospective study to evaluate effects of co-sleeping. The sentence has been changed a bit on line 256.

Discussion - Some comment re missing data would be helpful , 14 cases missing in gender , 25 cases in gestation and more than half cases had missing data on maternal smoking - how it may affect the reliability of the data esp with regards to smoking , although this is a known risk factor in sleep related SUDI. Sleeping position was missing in 35 cases. These are understandable given the retrospective nature of the study - so a comment may be useful to the reader

Answer;

Because this was a retrospective study, it was impossible to get all data without any lucks. A statement concerning this limitation was added into the revised text. The sentence was inserted into the first sentence of the first paragraph of Discussion (line 235 in the track changed text). Please also see lines 244-249 in the revised version.

Line 238 - Could the authors comment if the peak age of death at one month is related to the age group being studied to be 0-12 months of age as compared to other SUDI studies where the study group is from 2 to 12 months ?

Answer;

Good point! Other SUDI studies [4,24] showed the peak age to 2 months. As far as we read the articles, no apparent differences were found. However, we thought that the difference in peak age might be caused by that a particularly higher risk was evident among teenage mothers, and that more first-born infants were dead during 0–2 months of age. This message is replaced on lines 239-241.

Prevention issues - of the risk groups are not discussed much . Any comments on how the babies of later birth order might be at risk ?

Answer;

The authors are sorry for no idea about prevention issues to infants in later birth order, because we are not clinicians who care a number of alive patients. But according to the advice from the reviewer, we added a general sentence concerning prevention into line 278 of the track changed text.

Is there a concern about neglect if the duration of an infant seen alive is more than 5-6 hours for those found in the morning, and more than 2-3 hours for those found in the afternoon (since the parents or childminder would be awake then)? Is there a likely delay in reporting due to possible infanticide/ negligence in those reporting that last seen alive was 8 hours ? Or is it a non carer who was reporting his duration? Lack of this data could be a discussion point in your study re improvement in investigation by the social workers or police.

Issues related to DSI itself could be a discussion point on quality improvement in documentation and investigative process, were there any cases which could have been missed infanticide ?

Answer;

The authors absolutely agree with this comment by the reviewer. A striking finding was the prolonged time of discovery. We thought no apparent infanticide was involved in because DSI was performed by police officers, and pathologists have experiences to find out enforced events. But neglect was highly suspected for a couple of cases, particularly for which more than 10 hours was spent to find the unresponsive infant. A new paragraph was created in the Discussion section (line 266 of the track changed text). Please also see lines 281-285 in the revised version. Moreover, a sentence concerning this finding is added into the last part of Abstract (lines 48-49 in the track changed text).
