# Supplementary material for: Circumstances and factors of sleep-related sudden infancy deaths in Japan
Source: PLoS One. 2020 Aug 21;15(8):e0233253. doi: 10.1371/journal.pone.0233253 (PMC7444554; doi:10.1371/journal.pone.0233253)
Supplement: Attachment — Submitted filename: Responce to Reviwers.docx [file pone.0233253.s004.docx]

Reviewer #2:

Thank you very much for the detailed comments to our submission. The raised comments were helpful to revise the manuscript. The reply is described below. The answer to miscellaneous comments in the PDF file is indicated in the file as well. Alterations are indicated in the revised text in red.

1. I would suggest to authors if removing the words "during sleep" and from the objective would make the study different? SUDI by definition happens during sleep. It would make the methodology wording less confusing

Reply

The authors agree with the point that the reviewer suggested. According to the comment, the words "during sleep" have been replaced by "sleep-related" as the first submission was.

2. The methodology is not clear partly from the language, so i have made some suggestions to insert the phrases in the comments or exchange some words that are crossed out with those in the comments (if u place your mouse cursor over the speech icon) in the attached pdf file . Hope the authors can check if the suggestions are what they meant

Reply

Thank you for the detailed check to the text. We followed all the suggestions. Please see the revised version, and the PDF file.

3. The formatting of tables in the original submission are better, except that the early and late infancy columns have been removed

Reply

According to the suggestion, all Tables 1 to 3 are formatted like the original version, except for the removal of early and late columns.

4. Suggest that the 14% of the national population used in the calculation of the rates be displayed in the table instead of the whole national population birth data so that the denominator for the calculation of the SUDI rates are clear

Reply

The authors agree with what the reviewer pointed out. But the replacement in the column to the local population by multiplying 0.14 is unnatural for us. It is because only the total population is real. The area covered by the participants often overlaps to that of another institution. Each institution has a limited capacity for autopsy, so that police usually can choose the alternative one. The number of 0.14 is not strict at all, but actually tentative. We would like to show the total number as the last version was. Instead, a proviso is added into the bottom of the revised table with asterisk. Please see line 177.

5. More discussion on the limitations of the study due to the methodology can be added as given in the comments in the pdf

]

Reply

According to the advice, the statement concerning the limitations is transferred into the section of Discussion. Please see lines 294-295 and 310-312.
